# Supplementary material for: Discovery of S-217622, a Noncovalent Oral SARS-CoV-2 3CL Protease Inhibitor Clinical Candidate for Treating COVID-19
Source: J Med Chem. 2022 Mar 30;65(9):6499–512. doi: 10.1021/acs.jmedchem.2c00117 (PMC8982737; doi:10.1021/acs.jmedchem.2c00117)
Supplement: Supplementary file 1 — jm2c00117_si_001.pdf [file jm2c00117_si_001.pdf]

Supporting information

Discovery of S-217622, a Non-Covalent Oral SARS-CoV-2 3CL Protease Inhibitor Clinical Candidate for Treating COVID-19

Yuto Unoh[1]†, Shota Uehara[1]†, Kenji Nakahara[1]†, Haruaki Nobori[1], Yukiko Yamatsu[1], Shiho Yamamoto[1], Yuki Maruyama[1,2], Yoshiyuki Taoda[1], Koji Kasamatsu[1], Takahiro Suto[1], Kensuke Kouki[1], Atsufumi Nakahashi[1], Sho Kawashima[1], Takao Sanaki[1], Shinsuke Toba[1,2], Kentaro Uemura[1,2], Tohru Mizutare[1], Shigeru Ando[1], Michihito Sasaki[2], Yasuko Orba[2], Hirofumi Sawa[2], Akihiko Sato[1,2], Takafumi Sato[1], Teruhisa Kato[1], Yuki Tachibana[1]*

[1]Shionogi Pharmaceutical Research Center, 3-1-1 Futaba-cho, Toyonaka, Osaka, 561-0825, Japan

[2]International Institute for Zoonosis Control, Hokkaido University, Sapporo, 001-0020, Japan

*Corresponding author, E-mail: yuuki.tachibana@shionogi.co.jp (Yuki Tachibana)

†These authors contributed equally.

Contents

- Table S1. SAR studies of P1'/P2/P1 ligands……………………………..…………………..……..S2
- Table S2. The calculated IC_{50} values for S-217622 to SARS-CoV-2 3CL protease activity in each experiment……………………………………………………………………………………..S3
-
- Table S3. EC_{50} values of S-217622 and remdesivir on cytopathic effects in SARS-CoV-2 infected VeroE6/TMPRSS2 cells…………………………………………………………………..………...S3
- Table S4. CC_{50} values of S-217622 and remdesivir on cytopathic effects in VeroE6/TMPRSS2 cells……………………………………………………………………………………………S4
- Table S5. EC_{50} and EC_{90} values of S-217622 and remdesivir in SARS-CoV and MERS-CoV-2-infected VeroE6/TMPRSS2 cells, in HCoV-229E and HCoV-OC43-infected MRC-5 cells……….S4
- Table S6. *In vitro* safety profiles of S-217622……………………………………………………S4
- Table S7. Diffraction data and refinement statistics for SARS-CoV-2 $3CL^{pro}$ complexed with compound **1** and compound **3** (**S-217622**)………………………………………………………..S5
- Experimental procedures for the synthesis of compounds **13**-**21**……………………………….....S6
- ^{1}H-NMR and ^{13}C-NMR spectra for synthesized compounds……………………………….....S13
- HPLC traces of **1-3** (S-217622)…………………………………………………………….…...S33
- Experimental Procedures for *in vitro* safety…………………………………………...……..S37

Table S1. SAR studies of P1'/P2/P1 ligands

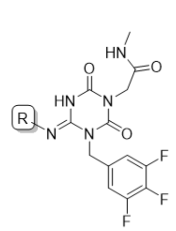

Compound	R	SARS-CoV-2 3CL^{pro} IC$_{50}$ (μM)
**1**	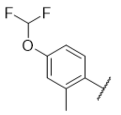	8.6
**13**	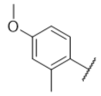	1.9
**14**	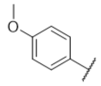	2.8
**15**	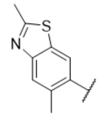	3.5
**2**	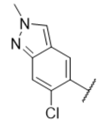	0.096

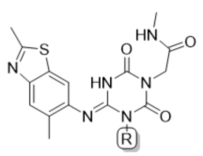

Compound	R	SARS-CoV-2 3CL^{pro} IC$_{50}$ (μM)
**15**	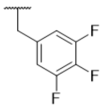	3.5
**16**	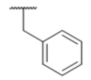	58
**17**	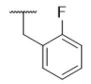	35
**18**	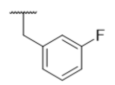	29
**19**	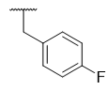	16

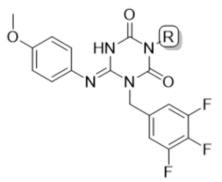

Compound	R	SARS-CoV-2 3CL^{pro} IC$_{50}$ (μM)
**14**	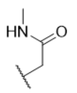	2.8
**20**	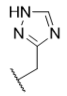	1.1
**21**	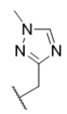	0.28

Table S2. The calculated IC_{50} values for S-217622 to SARS-CoV-2 3CL protease activity in each experiment.

Experiment	IC_{50} (μM)
Exp. No.1	0.0122
Exp. No.2	0.0130
Exp. No.3	0.0143
Mean	0.0132
Standard deviation	0.0011

Table S3. EC_{50} values of S-217622 and remdesivir on cytopathic effects in SARS-CoV-2 infected VeroE6/TMPRSS2 cells. [a]standard deviation. The mean and SD were calculated from three independent experiments.

Strains	Pango Lineage	EC_{50} (μM)			
		S-217622		Remdesivir	
		Mean	SD[a]	Mean	SD[a]
hCoV-19/Japan/TY/WK-521/2020	A	0.37	0.06	1.9	0.14
hCoV-19/Japan/QK002/2020	B.1.1.7	0.33	0.05	0.87	0.03
hCoV-19/Japan/QHN001/2020	B.1.1.7	0.31	0.07	0.97	0.14
hCoV-19/Japan/QHN002/2020	B.1.1.7	0.46	0.04	0.99	0.18
hCoV-19/Japan/TY7-501/2021	P.1	0.50	0.05	2.1	0.4
hCoV-19/Japan/TY7-503/2021	P.1	0.43	0.00	1.0	0.2
hCoV-19/Japan/TY8-612/2021	B.1.351	0.40	0.05	1.2	0.3
hCoV-19/Japan/TY11-927-P1/2021	B.1.617.2	0.41	0.01	1.6	0.2
hCoV-19/Japan/TY38-873/2021	B.1.1.529	0.29	0.05	1.1	0.3

Table S4. CC_{50} values of S-217622 and remdesivir against VeroE6/TMPRSS2 cells. [a]standard deviation. The mean and SD were calculated from three independent experiments.

Substance	CC_{50} (μM)				
	Exp.1	Exp.2	Exp.3	Mean	SD[a]
S-217622	>100	>100	>100	>100	-
Remdesivir	>100	>100	>100	>100	-

Table S5. EC_{50} and EC_{90} values of S-217622 and remdesivir in SARS-CoV and MERS-CoV-2-infected VeroE6/TMPRSS2 cells, in HCoV-229E and HCoV-OC43-infected MRC-5 cells. [a]standard deviation. The mean and SD were calculated from three independent experiments.

**Coronavirus Family**	**Cells**	**Assay**		**Efficacy (μM)**			
				**S-217622**		**Remdesivir**	
				**Mean**	**SD[a]**	**Mean**	**SD[a]**
SARS-CoV	VeroE6/TMPRSS2	CPE	EC_{50} (μM)	0.21	0.10	1.3	0.2
MERS-CoV	VeroE6/TMPRSS2	CPE	EC_{50} (μM)	1.4	0.2	1.5	0.2
HCoV-229E	MRC-5	CPE	EC_{50} (μM)	5.5	0.8	0.041	0.012
HCoV-OC43	MRC-5	RT-qPCR	EC_{90} (μM)	0.074	0.008	0.024	0.015

Table S6. *In vitro* safety profiles of S-217622

***In vitro* safety assays**	**Results**
hERG inhibition assay	>100 μM
Bacterial reverse mutation test (Ames test)	Negative
Micronucleus test	Negative
3T3 assay	No Phototoxicity

Table S7. Diffraction data and refinement statistics for SARS-CoV-2 3CL^{pro} complexed with compound **1** and compound **3** (**S-217622**).

a

	**3CL^{pro} – Compound 1**	**3CL^{pro} – Compound 3 (S-217622)**
**PDB code**	7VTH	7VU6
**Data collection**		
Space group	P21	P21
Cell dimensions		
a, b, c (Å)	44.41 54.27 114.50	55.47 99.23 58.88
α, β, γ (°)	90.00 99.42 90.00	90.00 108.05 90.00
Wavelength (Å)	1.54178	1.54178
Resolution (Å)	28.24 – 2.00(2.05 -2.00)	28.68 - 1.80(1.84 -1.80)
Completeness (%)	99.5 (98.9)	99.8(100.0)
R_{merge} *(%)*[a,b]	9.0(27.7)	6.0(42.9)
$I/\sigma(I)$ [a]	8.3(3.3)	20.5(2.6)
**Refinement**		
Resolution (Å)	112.952 – 2.001	55.986-1.800
No. of reflections	34366	52687
R_{work}/R_{free}	0.2050/0.2568	0.2241/0.2790
B factor (Å2)		
Protein	16.2	17.8
Ligand	27.4	14.3
Water	22.2	25.3
R.m.s deviations		
Bond length (Å)	0.0128	0.0175
Bond angles (°)	1.576	1.796
Ramachandran plot (%)		
Favored	97.2	97.5
Allowed	2.1	2.2
Outliers	0.7	0.3

Values in parentheses are for the highest resolution shell.

[b] $R_{merge} = \Sigma| I - \langle I \rangle |/\Sigma I$, where *I* is the intensity of observation *I* and $\langle I \rangle$ is the mean intensity of the reflection.

Scheme S1. Synthesis of compound 13

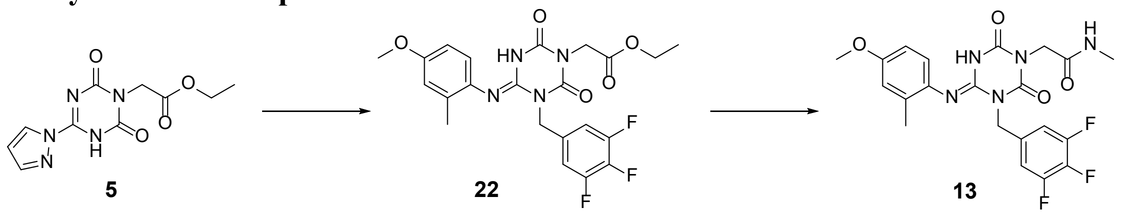

5 22 13

Ethyl (E)-2-{4-[(4-methoxy-2-methylphenyl)imino]-2,6-dioxo-3-(3,4,5-trifluorobenzyl)-1,3,5-triazinan-1-yl}acetate (22)

A mixture of **5** (100 mg, 0.377 mmol), *N*,*N*-diisopropylethylamine (0.079 mL, 0.452 mmol), and 5-(bromomethyl)-1,2,3-trifluorobenzene (0.055 mL, 0.415 mmol) in DMA (1 mL) was stirred at 60 °C for 1.5 h. The reaction mixture was cooled to room temperature and then 4-methoxy-2-methylaniline (0.053 mL, 0.415 mmol) was added. The mixture was stirred at 100 °C for 4 h. The reaction mixture was cooled to room temperature and quenched with saturated aqueous NH_4Cl. The aqueous layer was extracted with EtOAc. The organic layer was washed with H_2O and brine, dried over Na_2SO_4, and concentrated under reduced pressure. The residue was purified by silica gel column chromatography (n-hexane/EtOAc, gradient, 33-50% EtOAc) to afford **22** (96.3 mg, 53%) as a white solid. ^1H NMR (400 MHz, $CDCl_3$) δ 1.30 (3H, t, J = 7.2 Hz), 2.00 (3H, s), 3.79 (s, 3H), 4.25 (2H, q, J = 7.2 Hz), 4.58 (2H, s), 5.18 (2H, s), 6.65 (1H, d, J = 8.5 Hz), 6.75 (1H, dd, J = 8.5, 2.8 Hz), 6.80 (1H, d, J = 2.8 Hz), 7.20 (2H, dd, J = 8.1, 6.7 Hz). MS-ESI (m/z): 479 $[M + H]^+$.

(E)-2-{4-[(4-Methoxy-2-methylphenyl)imino]-2,6-dioxo-3-(3,4,5-trifluorobenzyl)-1,3,5-triazinan-1-yl}-N-methylacetamide (13)

To a stirred solution of **22** (91.8 mg, 0.192 mmol) in THF/MeOH (1.8 mL, v/v = 1/1) was added NaOH (1 M aqueous solution, 0.576 mL, 0.576 mmol) at room temperature. After stirring at room temperature overnight, the reaction mixture was quenched with 1 M aqueous HCl solution. The aqueous layer was extracted with EtOAc. The organic layer was washed with H_2O and brine, dried over $MgSO_4$, and concentrated under reduced pressure to afford a crude residue. The residue was dissolved in THF (1.7 mL), and then methylamine (2 M in THF, 0.142 mL, 0.283 mmol), *N*,*N*-diisopropylethylamine (0.066 mL, 0.377 mmol), and HATU (108 mg, 0.283 mmol) were added. After stirring at room temperature overnight, the reaction mixture was diluted with H_2O and EtOAc. The aqueous layer was extracted with EtOAc. The organic layer was washed with H_2O and brine, dried over $MgSO_4$, and concentrated under reduced pressure. The residue was purified by silica gel column chromatography ($CHCl_3$/MeOH, gradient, 0-10% MeOH). The collected fraction was recrystallized from *n*-hexane/EtOAc to afford **13** (57.0 mg, 64% in 2 steps) as a white solid. ^1H NMR (400 MHz, DMSO-d_6, DCl in D_2O) δ 1.91 (3H, s), 2.59 (3H, s), 3.74 (3H, s), 4.29 (2H, s), 5.32 (2H, s), 6.77-6.81 (2H, m), 6.98 (1H, d, J = 8.5 Hz), 7.38-7.42 (2H, m), 8.35 (1H, s). ^{13}C NMR (100 MHz, DMSO-d_6, DCl in D_2O) δ 17.50, 25.28, 43.91, 44.05, 55.15, 111.50 (dd, J = 16.5, 4.8 Hz), 111.57, 115.36, 128.14, 128.87, 133.12 (dt, J = 7.3, 4.4 Hz), 135.73, 137.80 (dt, J = 248.4, 15.2 Hz), 150.11 (ddd, J = 246.5, 9.9, 3.3 Hz), 150.95-151.15 (m), 151.05, 152.40, 157.79, 166.97. HRMS-ESI (m/z): $[M + H]^+$ calcd for $[C_{21}H_{21}F_3N_5O_4]^+$ 464.1540; found 464.1533; purity: 100% (LCMS).

Scheme S2. Synthesis of compounds 15, 17-19

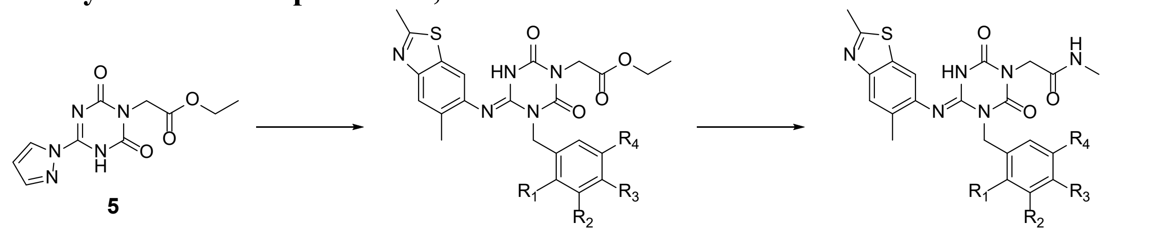

5

23 (R_1=H, R_2=F, R_3=F, R_4=F)
24 (R_1=F, R_2=H, R_3=H, R_4=H)
25 (R_1=H, R_2=F, R_3=H, R_4=H)
26 (R_1=H, R_2=H, R_3=F, R_4=H)

15 (R_1=H, R_2=F, R_3=F, R_4=F)
17 (R_1=F, R_2=H, R_3=H, R_4=H)
18 (R_1=H, R_2=F, R_3=H, R_4=H)
19 (R_1=H, R_2=H, R_3=F, R_4=H)

Ethyl 2-{4-[(2,5-dimethylbenzo[d]thiazol-6-yl)amino]-2,6-dioxo-3-(3,4,5-trifluorobenzyl)-3,6-dihydro-1,3,5-triazin-1(2H)-yl}acetate (23)

A mixture of **5** (1.00 g, 3.77 mmol), *N,N*-diisopropylethylamine (0.856 mL, 4.90 mmol), and 5-(bromomethyl)-1,2,3-trifluorobenzene (0.933 g, 4.15 mmol) in DMA (10 mL) was stirred at 60 °C for 1 h. 5-Bromomethyl-1,2,3-trifluorobenzene (0.255 g, 1.13 mmol) and *N,N*-diisopropylethylamine (0.197 mL, 1.13 mmol) were added to the mixture and the resulting mixture was stirred at 60 °C for 3 h. The reaction mixture was cooled to room temperature, and diluted with H_2O and EtOAc. The aqueous layer was extracted with EtOAc. The organic layer was washed with H_2O and brine, dried over Na_2SO_4, and concentrated under reduced pressure to afford a crude residue. The crude residue and 2,5-dimethyl-1,3-benzothiazol-6-amine (874 mg, 4.90 mmol) were disolved in DMA (7.7 mL) and stirred at 120 °C for 4 h. Then the reaction mixture was allowed to cool to room temperature. The mixture was diluted with H_2O and EtOAc and then the aqueous layer was extracted with EtOAc. The organic layer was washed with H_2O and brine, dried over Na_2SO_4, and concentrated under reduced pressure. The crude mixture was triturated with CH_2Cl_2/EtOAc and filtered to afford **23** (643 mg, 33% in 2 steps) as a beige solid. The filtrate was purified by silica gel column chromatography (*n*-hexane/EtOAc, 50% EtOAc) to afford **23** (239 mg, 12% over 2 steps) as a brown solid. ^{1}H NMR (400 MHz, $CDCl_3$) δ 1.30 (3H, t, *J* = 7.2 Hz), 2.13 (3H, s), 2.82 (3H, s), 4.26 (2H, q, *J* = 7.2 Hz), 4.58 (2H, s), 5.21 (2H, s), 7.16 (1H, s), 7.21 (2H, dd, *J* = 8.1, 6.6 Hz), 7.45 (1H, s), 7.80 (1H, s). MS-ESI (m/z): 520 $[M + H]^+$.

2-{4-[(2,5-Dimethylbenzo[d]thiazol-6-yl)amino]-2,6-dioxo-3-(3,4,5-trifluorobenzyl)-3,6-dihydro-1,3,5-triazin-1(2H)-yl}-N-methylacetamide (15)

15 was prepared from **23** in a manner similar to that for **13** in 45% yield in 2 steps. White solid. ^{1}H NMR (400 MHz, $CDCl_3$) δ 2.07 (3H, s), 2.81 (3H, s), 2.88 (3H, d, *J* = 4.5 Hz), 4.00-4.11 (2H, m), 5.17 (2H, s), 5.99-6.14 (1H, m), 7.11 (1H, m), 7.17-7.20 (2H, m), 7.68-7.71 (1H, m). ^{13}C NMR (100 MHz, $CDCl_3$) δ 18.30, 20.04, 26.49, 43.28, 44.99, 112.76, 113.20 (dd, *J* = 16.1, 5.9 Hz), 123.65, 129.50, 132.27-132.53 (m), 137.80, 134.21, 141.53, 148.38, 149.90, 150.49, 151.04 (ddd, *J* = 264.1, 10.3, 3.7 Hz), 166.36, 166.56. HRMS-ESI (m/z): $[M + H]^+$ calcd for $[C_{22}H_{20}F_3N_6O_3S]^+$ 505.1264; found 505.1260; purity: 100% (LCMS).

Ethyl 2-{4-[(2,5-dimethylbenzo[d]thiazol-6-yl)amino]-3-(2-fluorobenzyl)-2,6-dioxo-3,6-dihydro-1,3,5-triazin-1(2H)-yl}acetate (24)

24 was prepared from **5** in a manner similar to that for **22** as a crude product. MS-ESI (m/z): 484 $[M + H]^+$.

2-{4-[(2,5-Dimethylbenzo[d]thiazol-6-yl)amino]-3-(2-fluorobenzyl)-2,6-dioxo-3,6-dihydro-1,3,5-triazin-1(2H)-yl}-N-methylacetamide (17)

17 was prepared from **24** in a manner similar to that for **13** in 26% yield in 4 steps. White solid. ^{1}H NMR (400 MHz, DMSO-d_6, DCl in D_2O) δ 2.01 (3H, s), 2.59 (3H, s), 2.81 (3H, s), 4.29 (2H, s), 5.32 (2H, s), 7.20-7.26 (2H, m), 7.34-7.41 (2H, m), 7.59 (1H, s), 7.74 (1H, s). ^{13}C NMR (100 MHz, DMSO-d_6, DCl in D_2O) δ 18.19, 19.78, 25.81, 44.18, 115.71 (d, J = 20.5 Hz), 117.63-117.98 (m), 122.49, 123.62 (d, J = 13.9 Hz), 124.92 (d, J = 2.9 Hz), 128.02 (d, J = 4.4 Hz), 129.60 (d, J = 8.1 Hz), 132.46, 132.66, 149.71, 150.90, 151.11, 159.11, 161.53, 167.26, 167.34, 167.82. HRMS-ESI (m/z): $[M + H]^+$ calcd for $[C_{22}H_{22}FN_6O_3S]^+$ 469.1453; found 469.1446; purity: 100% (LCMS).

Ethyl 2-{4-[(2,5-dimethylbenzo[d]thiazol-6-yl)amino]-3-(3-fluorobenzyl)-2,6-dioxo-3,6-dihydro-1,3,5-triazin-1(2H)-yl}acetate (25)

25 was prepared from **5** in a manner similar to that for **22** as a crude product. MS-ESI (m/z): 484 $[M + H]^+$.

2{4-[(2,5-Dimethylbenzo[d]thiazol-6-yl)amino]-3-(3-fluorobenzyl)-2,6-dioxo-3,6-dihydro-1,3,5-triazin-1(2H)-yl}-N-methylacetamide (18)

18 was prepared from **25** in a manner similar to that for **13** in 28% yield in 4 steps. White solid. ^{1}H NMR (400 MHz, DMSO-d_6, DCl in D_2O) δ 1.97 (3H, s), 2.59 (3H, s), 2.81 (3H, s), 4.30 (2H, s), 5.32 (2H, s), 7.11-7.16 (1H, m), 7.24-7.28 (2H, m), 7.40-7.46 (1H, m), 7.60 (1H, s), 7.73 (1H, s). ^{13}C NMR (100 MHz, DMSO-d_6, DCl in D_2O) δ 18.13, 19.95, 25.81, 44.19, 44.27, 114.17 (d, J = 22.0 Hz), 114.50 (d, J = 20.5 Hz), 118.07, 122.60, 123.27 (d, J = 2.9 Hz), 130.84 (d, J = 8.1 Hz), 132.49, 132.94, 139.59 (d, J = 7.3 Hz), 150.07, 151.14, 151.28, 161.51, 163.93, 167.33, 167.41, 167.71.
HRMS-ESI (m/z): $[M + H]^+$ calcd for $[C_{22}H_{22}FN_6O_3S]^+$ 469.1453; found 469.1446; purity: 100% (LCMS).

Ethyl 2-{4-[(2,5-dimethylbenzo[d]thiazol-6-yl)amino)]-3-(4-fluorobenzyl)-2,6-dioxo-3,6-dihydro-1,3,5-triazin-1(2H)-yl}acetate (26)

26 was prepared from **5** in a manner similar to that for **23** as a crude product. MS-ESI (m/z): 484 $[M + H]^+$.

2-{4-[(2,5-Dimethylbenzo[d]thiazol-6-yl)amino]-3-(4-fluorobenzyl)-2,6-dioxo-3,6-dihydro-1,3,5-triazin-1(2H)-yl}-N-methylacetamide (19)

19 was prepared from **26** in a manner similar to that for **13** in 32% yield in 4 steps. White solid. ^{1}H NMR (400 MHz, DMSO-d_6, DCl in D_2O) ^{1}H NMR (400 MHz, DMSO-d_6, DCl in D_2O) δ 1.98 (3H, s), 2.59 (3H, s), 2.81 (3H, s), 4.30 (2H, s), 5.29 (2H, s), 7.18-7.24 (2H, m), 7.45-7.49 (2H, m), 7.62 (1H, s), 7.74 (1H, s). ^{13}C NMR (100 MHz, DMSO-d_6, DCl in D_2O) δ 18.16, 19.81, 25.81, 44.22, 44.92, 115.62 (d, J = 21.3 Hz), 118.30, 122.53, 129.59 (d, J = 8.1 Hz), 132.74 (d, J = 2.9 Hz), 132.86, 149.94, 151.17, 151.38, 160.71, 163.12, 167.32, 167.40, 167.94. HRMS-ESI (m/z): $[M + H]^+$ calcd for $[C_{22}H_{22}FN_6O_3S]^+$ 469.1453; found 469.1446; purity: 100% (LCMS).

Scheme S3. Synthesis of compound 14

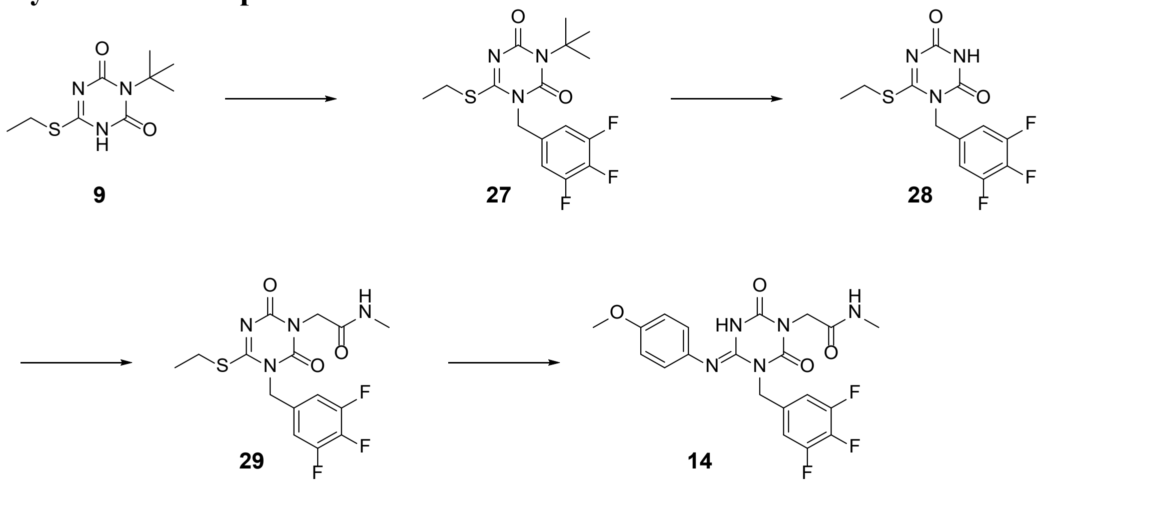

3-(tert-Butyl)-6-(ethylthio)-1-(3,4,5-trifluorobenzyl)-1,3,5-triazine-2,4(1H,3H)-dione (27)

27 was prepared from **9** in a manner similar to that for **10** in 93% yield. White solid. ^{1}H NMR (400 MHz, DMSO-d_6) δ 1.26 (3H, t, *J* = 7.2 Hz), 1.58 (9H, s), 3.07 (2H, q, *J* = 7.2 Hz), 4.97 (2H, s), 7.33 (2H, dd, *J* = 8.7, 6.9 Hz). MS-ESI (m/z): 374 $[M + H]^+$.

6-(Ethylthio)-1-(3,4,5-trifluorobenzyl)-1,3,5-triazine-2,4(1H,3H)-dione (28)

28 was prepared from **27** in a manner similar to that for **11** in 95% yield. White solid. ^{1}H NMR (400 MHz, $CDCl_3$) δ 1.39 (3H, t, *J* = 7.3 Hz), 3.26 (2H, q, *J* = 7.3 Hz), 5.05 (2H, s), 7.03 (2H, dd, *J* = 7.8, 6.4 Hz), 8.43 (1H, s). MS-ESI (m/z): 318 $[M + H]^+$.

2-[4-(Ethylthio)-2,6-dioxo-3-(3,4,5-trifluorobenzyl)-3,6-dihydro-1,3,5-triazin-1(2H)-yl]-N-methylacetamide (29)

A mixture of **28** (1.00 g, 3.15 mmol), 2-chloro-*N*-methylacetamide (441 mg, 4.10 mmol), and potassium carbonate (871 mg, 6.30 mmol) in DMF (10 mL) was stirred at 50 °C for 4 h. The reaction mixture was allowed to cool to room temperature and diluted with aqueous citric acid solution. The aqueous layer was extracted with EtOAc. The organic layer was washed with H_2O and brine, dried over Na_2SO_4, and concentrated under reduced pressure. The residue was triturated with n-hexane/diisopropyl ether/ethyl acetate to afford **29** (757 mg, 62%) as a white solid. ^{1}H NMR (400 MHz, $CDCl_3$) δ 1.39 (3H, t, *J* = 7.4 Hz), 2.87 (3H, d, *J* = 4.9 Hz) 3.25 (2H, q, *J* = 7.4 Hz), 4.56 (2H, s), 5.06 (2H, s), 5.68 (1H, s) 7.03 (2H, dd, *J* = 7.7, 6.5 Hz). MS-ESI (m/z): 389 $[M + H]^+$.

(E)-2-{4-[(4-Methoxyphenyl)imino]-2,6-dioxo-3-(3,4,5-trifluorobenzyl)-1,3,5-triazinan-1-yl}-N-methylacetamide (14)

A mixture of **29** (40 mg, 0.103 mmol), 4-methoxyaniline (17.0 mg, 0.134 mmol) and AcOH (118 µL, 2.06 mmol) in *tert*-butanol (0.80 mL) was stirred at 100 °C for 4 h. After cooling to room temperature, the mixture was concentrated under reduced pressure. The residue was purified by silica gel column chromatography (EtOAc/MeOH, gradient, 0-20% MeOH) to afford **14** (29.7 mg, 64%) as a white solid. ^{1}H NMR (400 MHz, DMSO-d_6, DCl in D_2O) δ 2.58 (3H, s), 3.75 (3H, s), 4.28 (2H, s), 5.33 (2H, s), 6.93 (2H, d, *J* = 8.8 Hz), 7.20 (2H, d, *J* = 8.8 Hz), 7.39 (2H, dd, *J* = 8.5, 7.0 Hz). ^{13}C-NMR (DMSO-d_6, DCl in D_2O) δ 25.79, 44.52, 44.59, 55.74, 111.94 (dd, *J* = 16.1, 5.1 Hz), 114.25, 126.98, 130.68, 133.58-133.77 (m), 135.73, 138.31 (dt, *J* = 248.7, 15.4 Hz), 150.62 (ddd, *J* = 247.2, 9.5, 3.7 Hz), 151.62,

152.05-152.16 (m), 153.13, 157.54, 167.54. HRMS-ESI (m/z): $[M + H]^+$ calcd for $[C_{20}H_{19}F_3N_5O_4]^+$ 450.1384; found 450.1375; purity: 98% (LCMS).

Scheme S4. Synthesis of compound 16

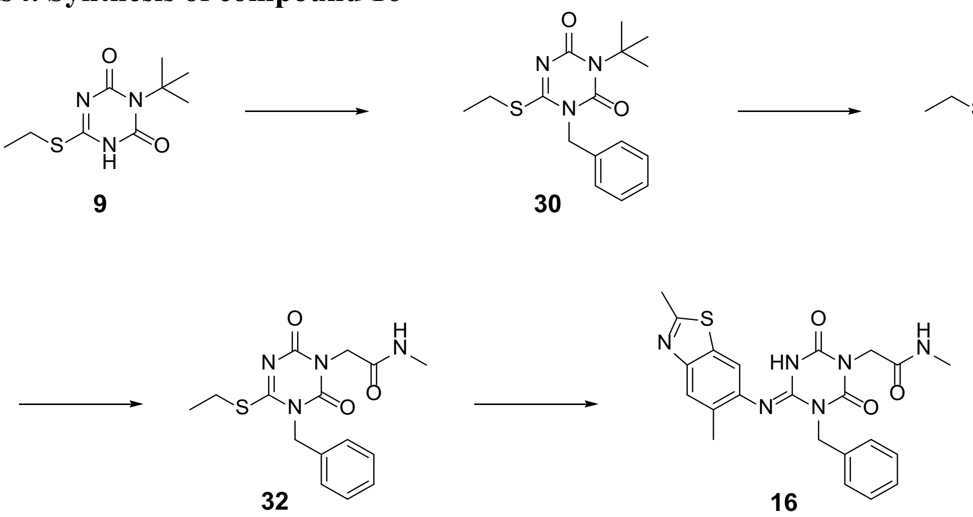

1-Benzyl-3-(tert-butyl)-6-(ethylthio)-1,3,5-triazine-2,4(1H,3H)-dione (30)

30 was prepared from **9** in a manner similar to that for **10**, and the crude mixture was used for the next reaction without further purification.

1-Benzyl-6-(ethylthio)-1,3,5-triazine-2,4(1H,3H)-dione (31)

31 was prepared from a crude mixture of **30** in a manner similar to that for **11** in 88% yield in 2 steps. White solid. ^{1}H NMR (300 MHz, DMSO-d_6) δ 1.25 (3H, t, J = 7.2 Hz), 3.08 (2H, q, J = 7.2 Hz), 5.40 (2H, s), 7.25-7.39 (5H, m), 11.60 (1H, s). MS-ESI (m/z): 264 $[M + H]^+$.

2-[3-Benzyl-4-(ethylthio)-2,6-dioxo-3,6-dihydro-1,3,5-triazin-1(2H)-yl]-N-methylacetamide (32)

32 was prepared from **31** in a manner similar to that for **29** in 71% yield. White solid. ^{1}H NMR (400 MHz, $CDCl_3$) δ 1.36 (3H, t, J = 7.4 Hz), 2.86 (3H, d, J = 4.9 Hz), 3.22 (2H, q, J = 7.4 Hz), 4.57 (2H, s), 5.16 (2H, s), 5.69 (1H, br s), 7.31-7.39 (5H, m). MS-ESI (m/z): 335 $[M + H]^+$.

(E)-2-(3-Benzyl-4-((2,5-dimethylbenzo[d]thiazol-6-yl)imino)-2,6-dioxo-1,3,5-triazinan-1-yl)-N-methylacetamide (16)

16 was prepared from **32** in a manner similar to that for **14** in 21% yield. White solid. ^{1}H NMR (400 MHz, DMSO-d_6, DCl in D_2O) δ 1.96 (3H, s), 2.59 (3H, s), 2.81 (3H, s), 4.31 (2H, s), 5.32 (2H, s), 7.29-7.32 (1H, m), 7.36-7.42 (4H, m), 7.64 (1H, s), 7.74 (1H, s). ^{13}C NMR (100 MHz, DMSO-d_6, DCl in D_2O) ^{13}C NMR (100 MHz, DMSO-d_6, DCl in D_2O) δ 17.63, 19.30, 25.30, 43.69, 45.03, 118.10, 122.00, 126.74, 127.26, 128.37, 132.31, 132.39, 135.96, 149.52, 150.70, 151.08, 166.81, 166.89, 167.55. HRMS-ESI (m/z): $[M + H]^+$ calcd for $[C_{22}H_{23}N_6O_3S]^+$ 451.1547; found 451.1537; purity: 98% (LCMS).

Scheme S5. Synthesis of compound 20

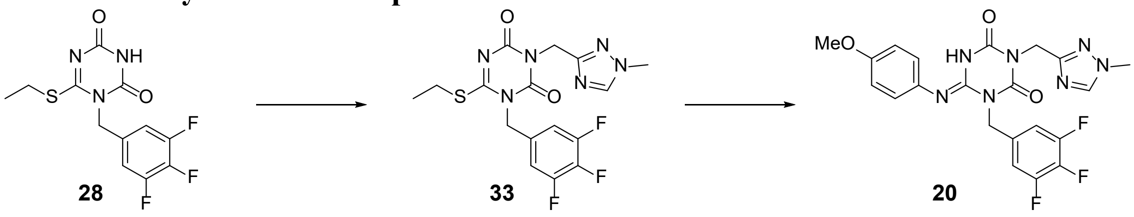

6-(Ethylthio)-3-[(1-methyl-1H-1,2,4-triazol-3-yl)methyl]-1-(3,4,5-trifluorobenzyl)-1,3,5-triazine-2,4(1H,3H)-dione (33)

A mixture of **28** (100 mg, 0.315 mmol), 3-(chloromethyl)-1-methyl-1*H*-1,2,4-triazole (45.6 mg, 0.347 mmol), and potassium carbonate (56.6 mg, 0.410 mmol) in DMF (1.0 mL) was stirred at 60 °C for 2 h. The reaction mixture was allowed to cool to room temperature and diluted with aqueous NH_4Cl solution. The aqueous layer was extracted with EtOAc, and the organic layer was washed with H_2O, dried over Na_2SO_4, and concentrated under reduced pressure to afford a crude product of **33** (109 mg) as a white solid. ^{1}H-NMR ($CDCl_3$) δ: 1.38 (3H, t, *J* = 7.4 Hz), 3.25 (2H, q, *J* = 7.4 Hz), 3.87 (3H, s), 5.09 (2H, s), 5.24 (2H, s), 7.02-7.06 (2H, m), 7.94 (1H, s). MS-ESI (m/z): 413 $[M + H]^+$.

(E)-6-((4-Methoxyphenyl)imino)-3-((1-methyl-1H-1,2,4-triazol-3-yl)methyl)-1-(3,4,5-trifluorobenzyl)-1,3,5-triazinane-2,4-dione (20)

After a mixture of **33** (50 mg, 0.121 mmol) and 4-methoxyaniline (22.4 mg, 0.182 mmol) in *tert*-butanol (0.50 mL) was stirred at 100 °C for 4 h, the reaction mixture was allowed to cool to room temperature. The mixture was concentrated under reduced pressure. The residue was purified by silica gel column chromatography (EtOAc/MeOH, gradient, 0-10% MeOH) to afford **20** (26.5 mg, 39% in 2 steps) as a white solid. ^{1}H-NMR (DMSO-d_6, DCl in D_2O) δ: 3.75 (3H, s), 3.94 (3H, s), 5.10 (2H, s), 5.44 (2H, s), 6.93 (2H, d, *J* = 8.5 Hz), 7.25 (2H, d, *J* = 8.5 Hz), 7.42-7.44 (2H, m), 9.36 (1H, s). ^{13}C-NMR (DMSO-d_6, DCl in D_2O) δ: 37.1, 37.8 , 44.0 , 55.2, 111.5 (dd, *J* = 16.1, 5.1 Hz), 113.7, 126.6, 129.7, 132.9 (dt, *J* = 4.4, 8.1 Hz), 137.8 (dt, *J* = 248.7, 15.4 Hz), 143.4, 150.1 (ddd, *J* = 247.2, 8.8, 3.7 Hz), 151.0, 152.2, 152.5, 155.2, 157.1. HRMS-ESI (m/z): $[M + H]^+$ calcd for $[C_{21}H_{19}F_3N_7O_3]^+$474.1496 ; found 474.1489; purity: 100% (LCMS).

Scheme S6. Synthesis of compound 21

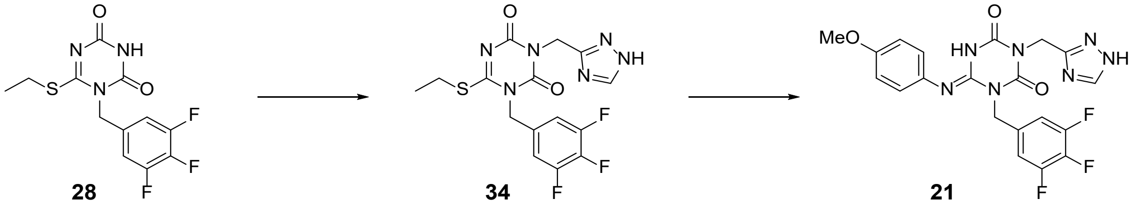

3-[(1H-1,2,4-Triazol-3-yl)methyl]-6-(ethylthio)-1-(3,4,5-trifluorobenzyl)-1,3,5-triazine-2,4(1H,3H)-dione (34)

To a solution of **28** (2.5 g, 7.88 mmol) in 1,4-dioxane (25 mL) was added (1-trityl-1*H*-1,2,4-triazol-3-yl)methanol (3.23 g, 9.46 mmol), triphenylphosphine (2.48 g, 9.46 mmol) and diisopropyl azodicarboxylate (1.84 mL, 9.46 mmol) at 0 °C. After stirring for 2 h at the same temperature, the reaction mixture was concentrated under reduced pressure. The residue was dissolved in CH_2Cl_2 (28.5 mL) and treated with TFA (6.85 ml, 89 mmol) at 0 °C, and the reaction mixture was allowed to warm to room temperature. After stirring for 4 h, aqueous $NaHCO_3$ solution was added, and the aqueous layer was extracted with $CHCl_3$. The organic layer was washed with brine, dried over $MgSO_4$, and concentrated under reduced pressure. The residue was purified by silica gel column chromatography (EtOAc/MeOH, gradient, 0-10% MeOH) to afford **34** (1.55 g, 44%) as a white solid. ^{1}H-NMR ($CDCl_3$) δ: 1.40 (3H, t, J = 7.4 Hz), 3.26 (2H, q, J = 7.4 Hz), 5.07 (2H, s), 5.30 (2H, s), 7.04 (2H, t, J = 7.0 Hz), 8.09 (1H, s). MS-ESI (m/z): 399 $[M + H]^+$.

(E)-3-((1H-1,2,4-triazol-3-yl)methyl)-6-((4-methoxyphenyl)imino)-1-(3,4,5-trifluorobenzyl)-1,3,5-triazinane-2,4-dione (21)

To a mixture of **34** (200 mg, 0.502 mmol) and 4-methoxyaniline (93 mg, 0.753 mmol) in acetic acid (0.431 mL, 7.53 mmol) was added *tert*-butanol (2 mL), and stirred at 100 °C for 5 h. Then the reaction mixture was allowed to cool to room temperature and diluted with aqueous $NaHCO_3$ solution. The aqueous layer was extracted with EtOAc. The organic layer was washed with brine, dried over Na_2SO_4, and concentrated under reduced pressure. The residue was purified by silica gel column chromatography (EtOAc/MeOH, gradient, 0-5% MeOH) to afford **21** (188 mg, 82%) as a white solid. ^{1}H NMR (400 MHz, DMSO-d_6, DCl in D_2O) δ 3.75 (3H, s), 5.18 (2H, s), 5.38 (2H, s), 6.93 (2H, d, J = 8.9 Hz), 7.24 (2H, d, J = 8.5 Hz), 7.41-7.47 (2H, m), 9.19 (1H, s). ^{13}C-NMR (DMSO-d_6, DCl in D_2O) δ: 36.7, 43.6, 54.7, 110.9 (dd, J = 16.5, 5.5 Hz), 113.2, 126.1, 129.4, 132.4 (dt, J = 7.5, 4.6 Hz), 137.3 (dt, J = 248.0, 15.4 Hz), 143.2, 149.6 (ddd, J = 243.6, 9.5, 3.7 Hz), 150.6, 151.6, 152.0, 152.5, 156.6. HRMS-ESI (m/z): $[M + H]^+$ calcd for $[C_{20}H_{17}F_3N_7O_3]^+$ 460.1339; found 460.1333; purity: 100% (LCMS).

^{1}H-NMR and ^{13}C-NMR spectra for synthesized compounds

^{1}H NMR spectra of **5** in DMSO-d_6

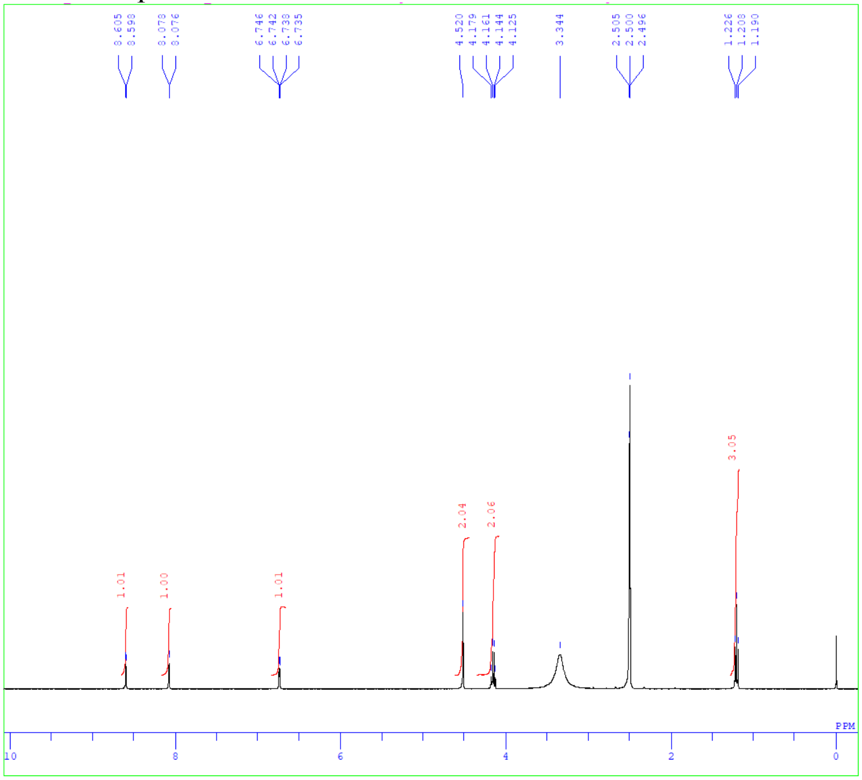

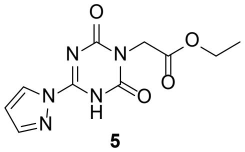

5

^{13}C NMR spectra of **5** in DMSO-d_6

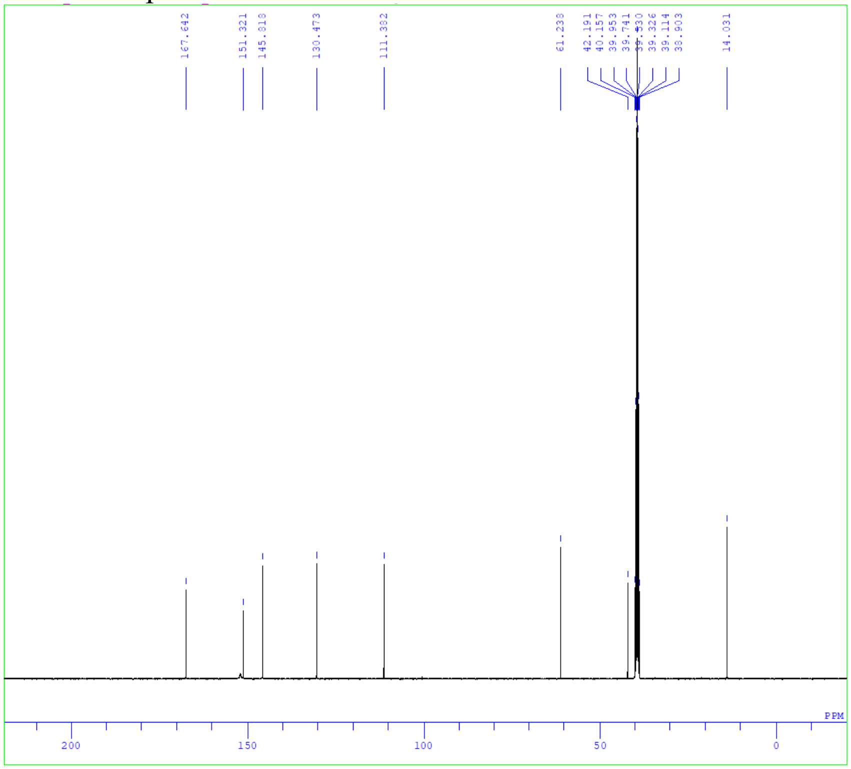

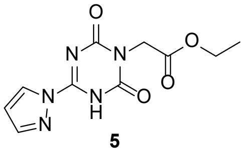

5

^{1}H NMR spectra of **7** in $CDCl_3$

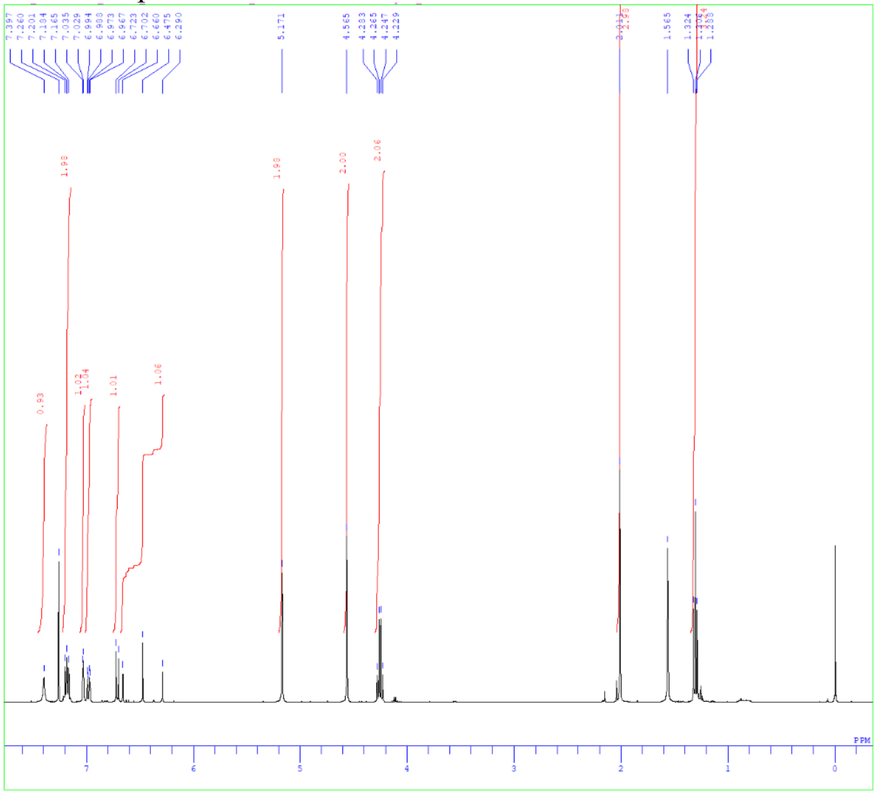

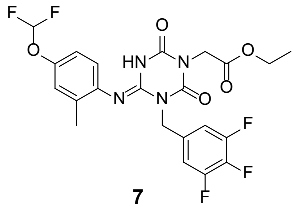

7

^{13}C NMR spectra of **7** in $CDCl_3$

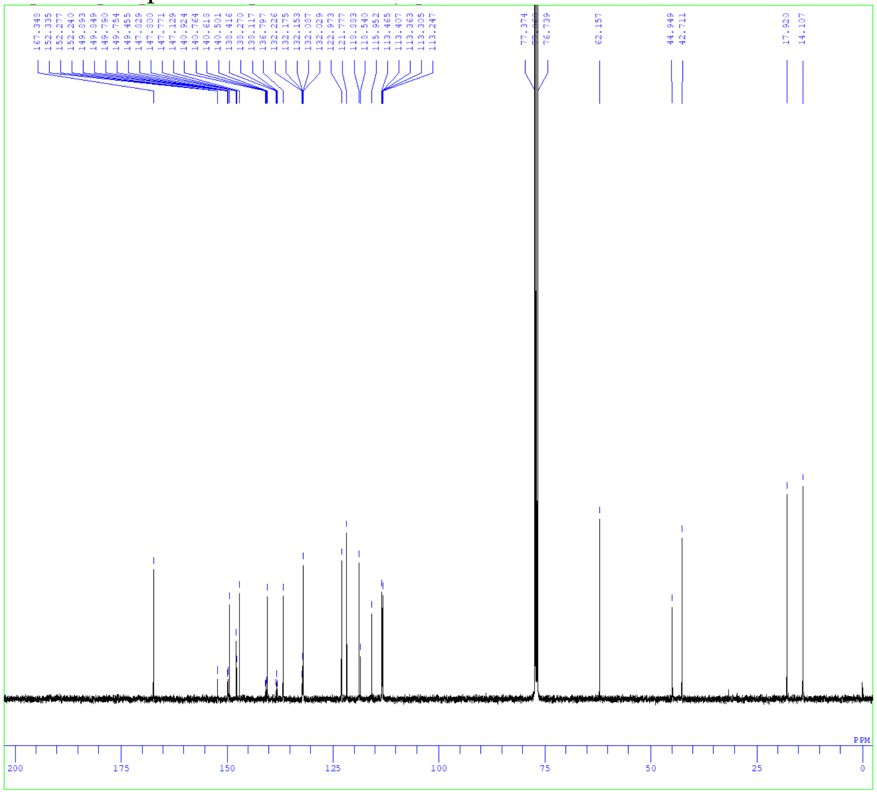

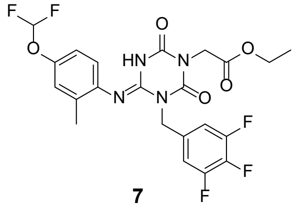

7

^{1}H NMR spectra of **1** in $CDCl_3$

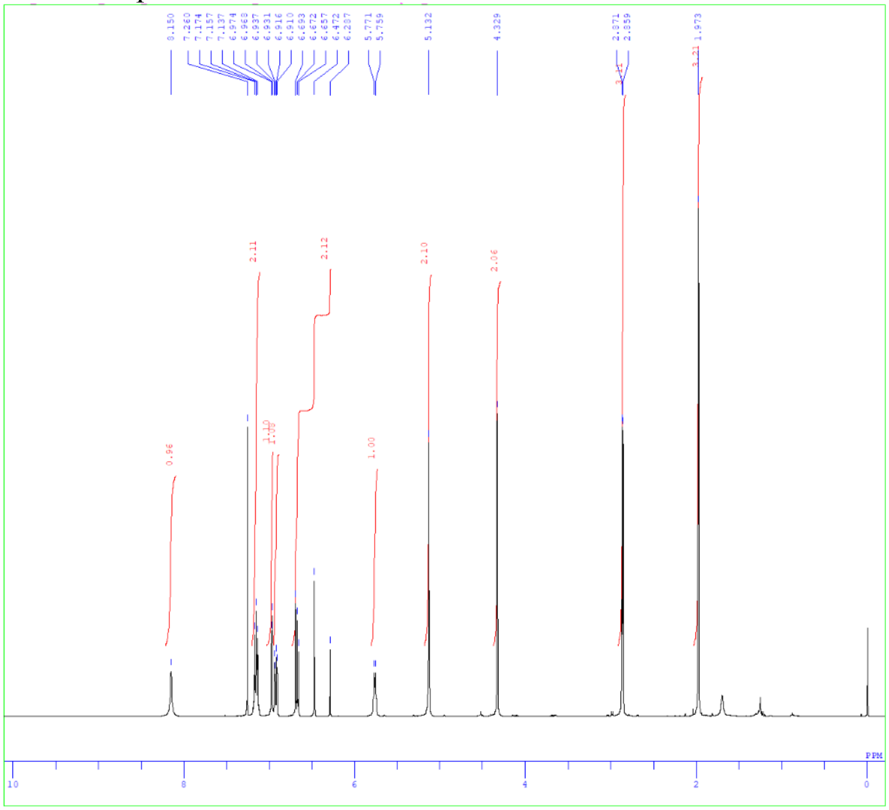

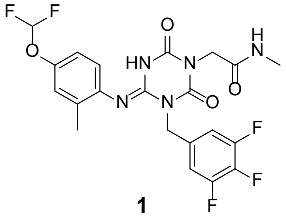

1

^{13}C NMR spectra of **1** in $CDCl_3$

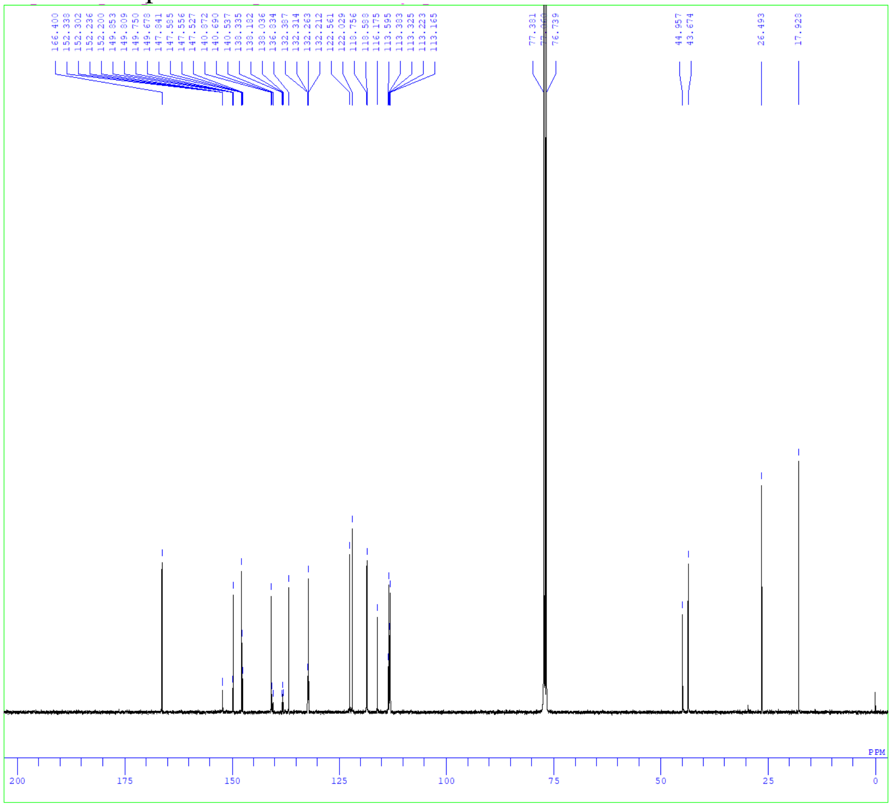

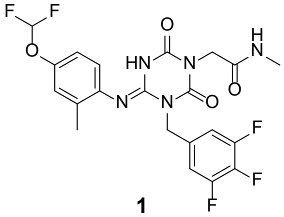

1

^{1}H NMR spectra of **8** in DMSO-d_6 with DCl in D_2O

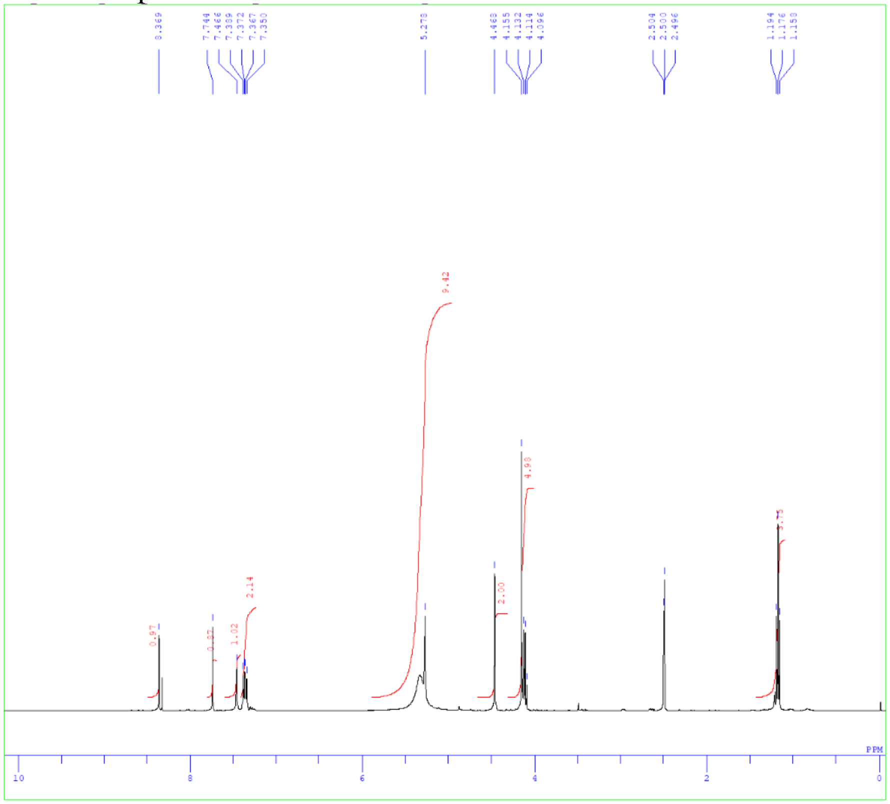

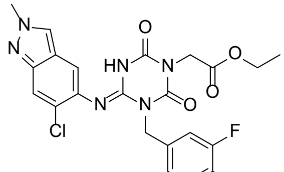

8

^{13}C NMR spectra of **8** in DMSO-d_6 with DCl in D_2O

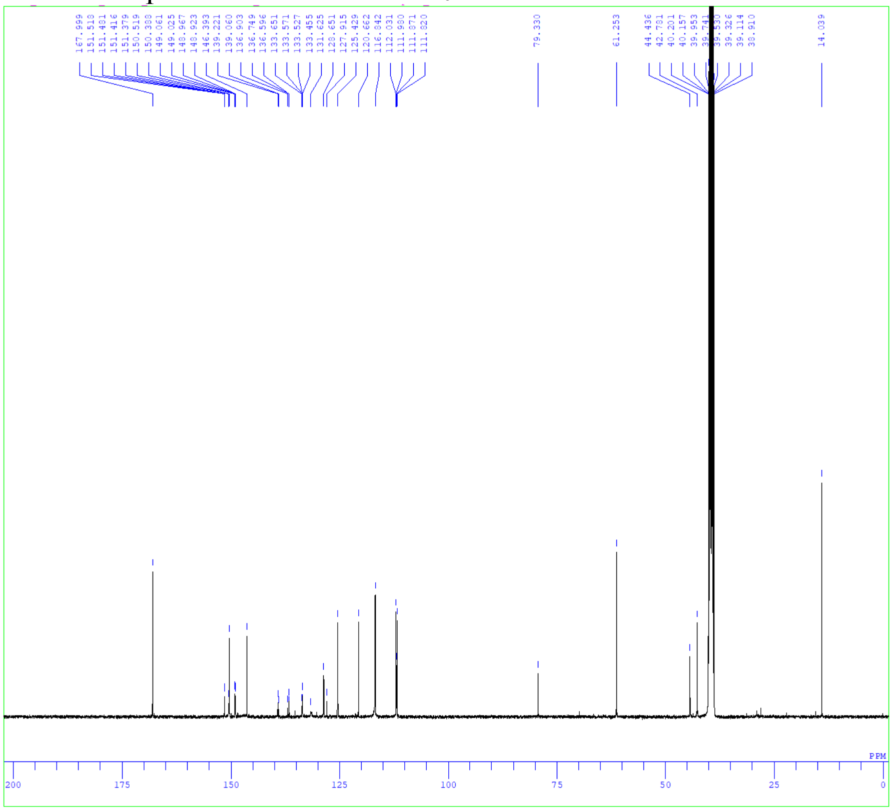

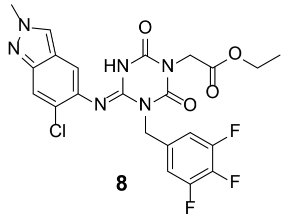

8

^{1}H NMR spectra of **2** in DMSO-d_6 with DCl in D_2O

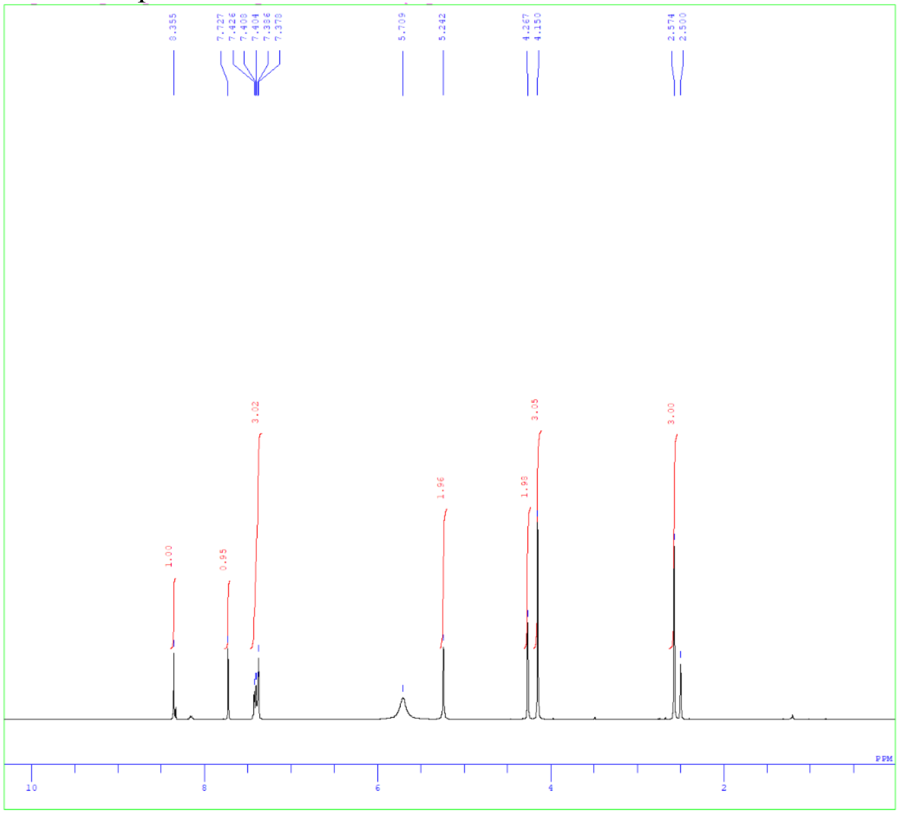

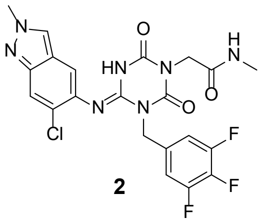

2

^{13}C NMR spectra of **2** in DMSO-d_6 with DCl in D_2O

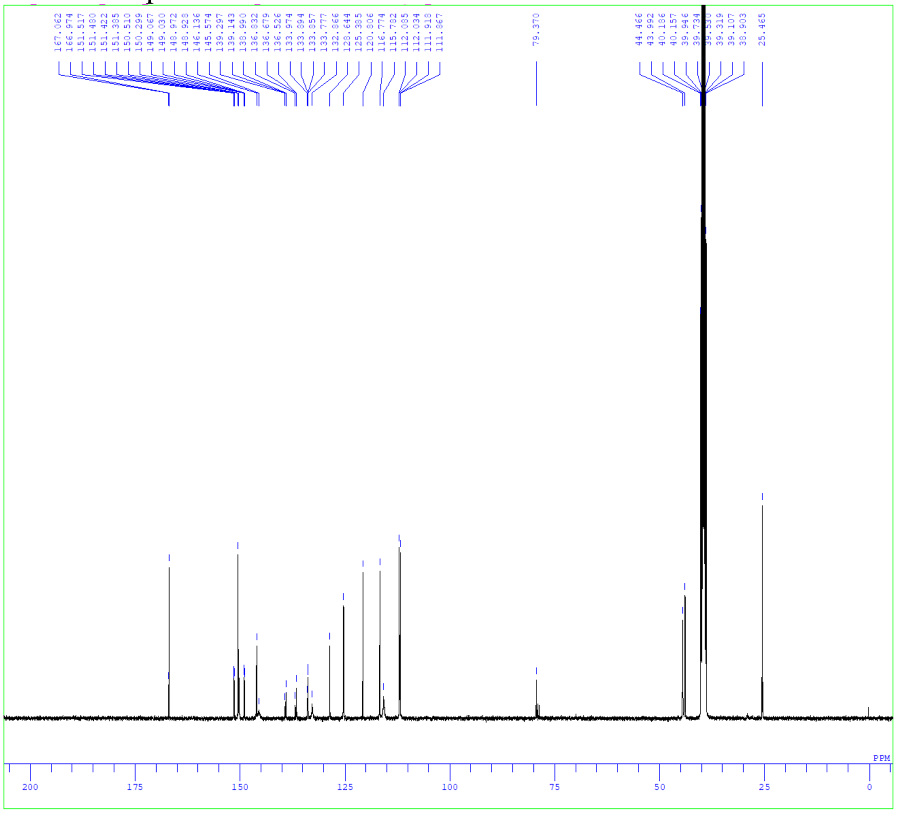

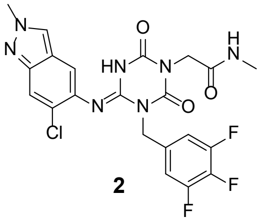

2

^{1}H NMR spectra of **9** in $CDCl_3$

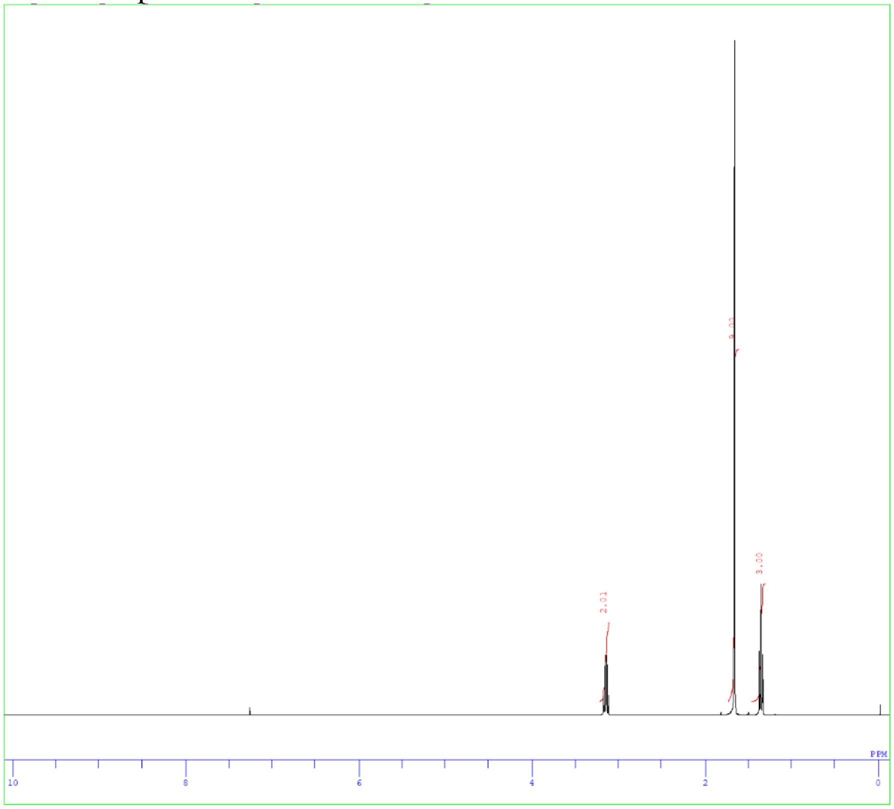

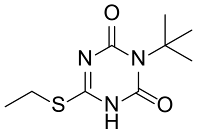

9

^{13}C NMR spectra of **9** in $CDCl_3$

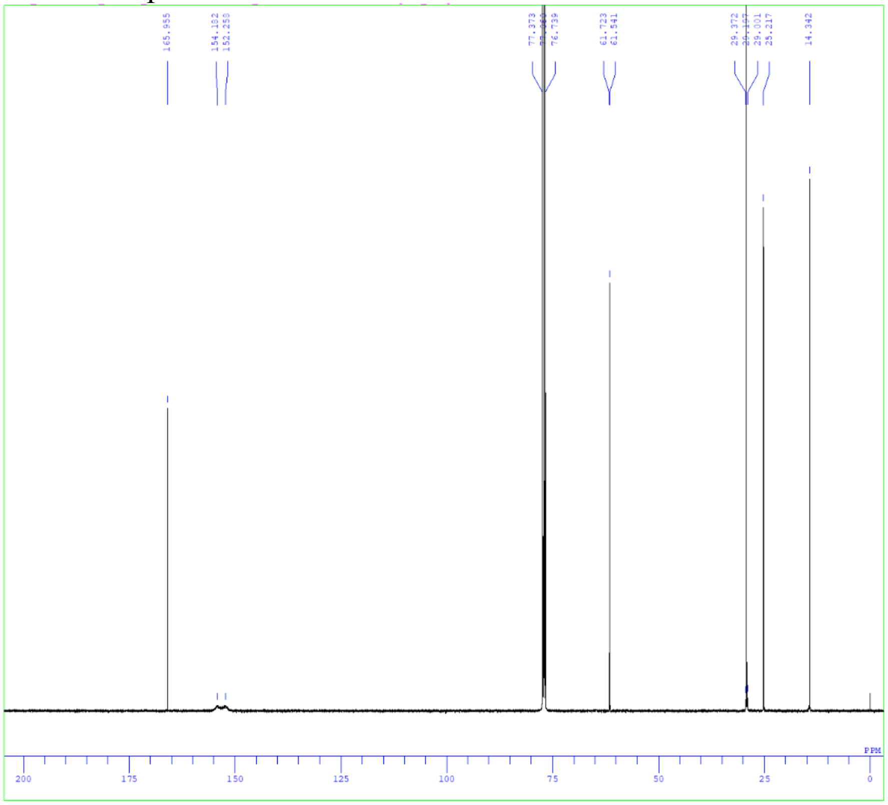

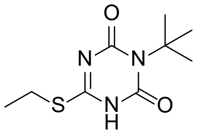

9

^{1}H NMR spectra of **10** in $CDCl_3$

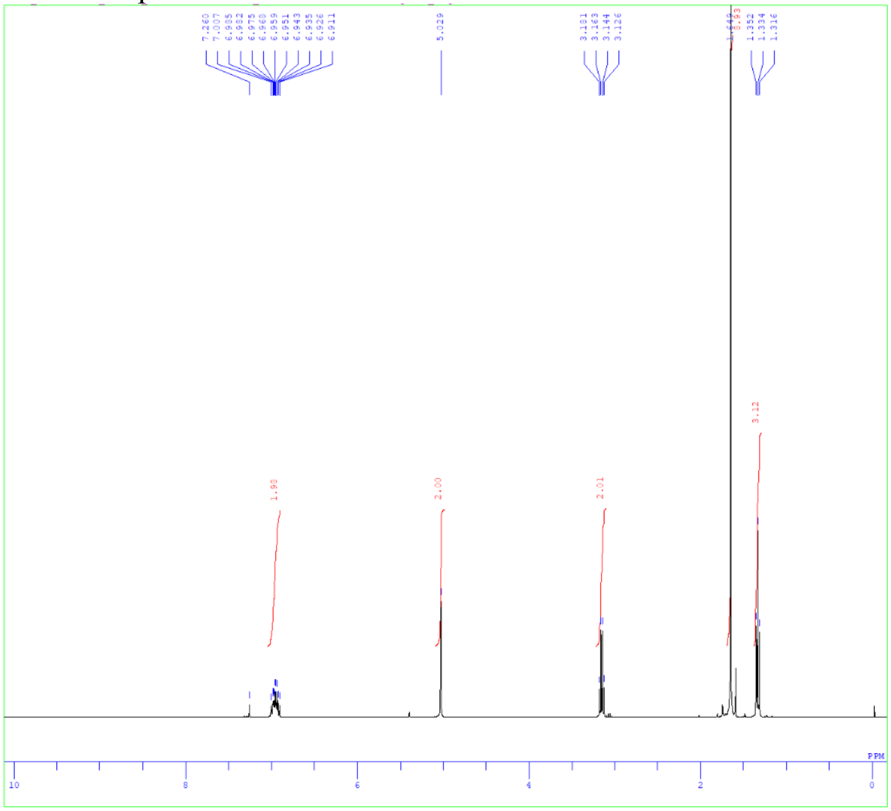

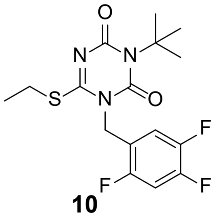

10

^{13}C NMR spectra of **10** in $CDCl_3$

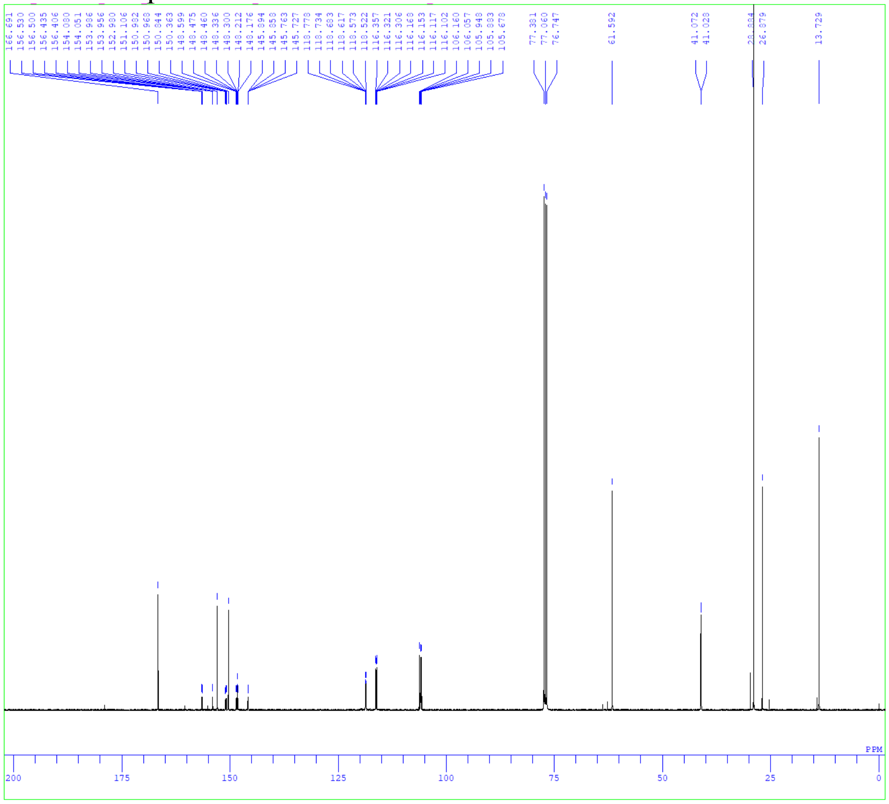

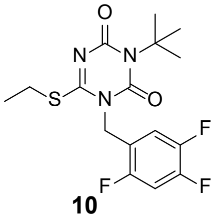

10

^{1}H NMR spectra of **11** in $CDCl_3$

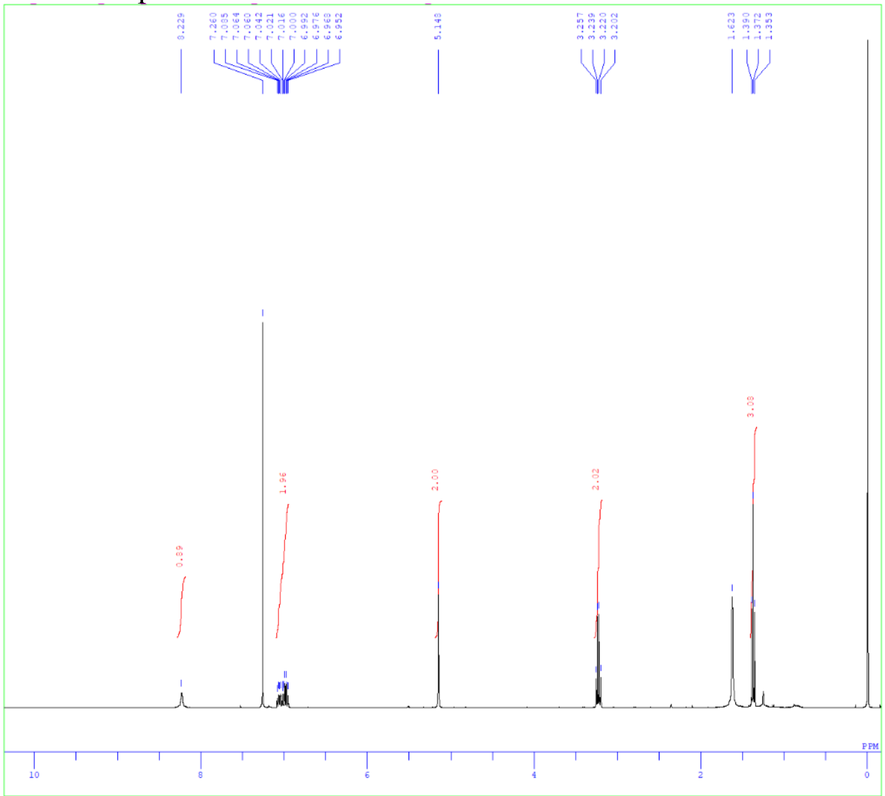

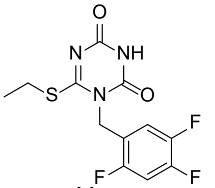

11

^{13}C NMR spectra of **11** in DMSO-d_6

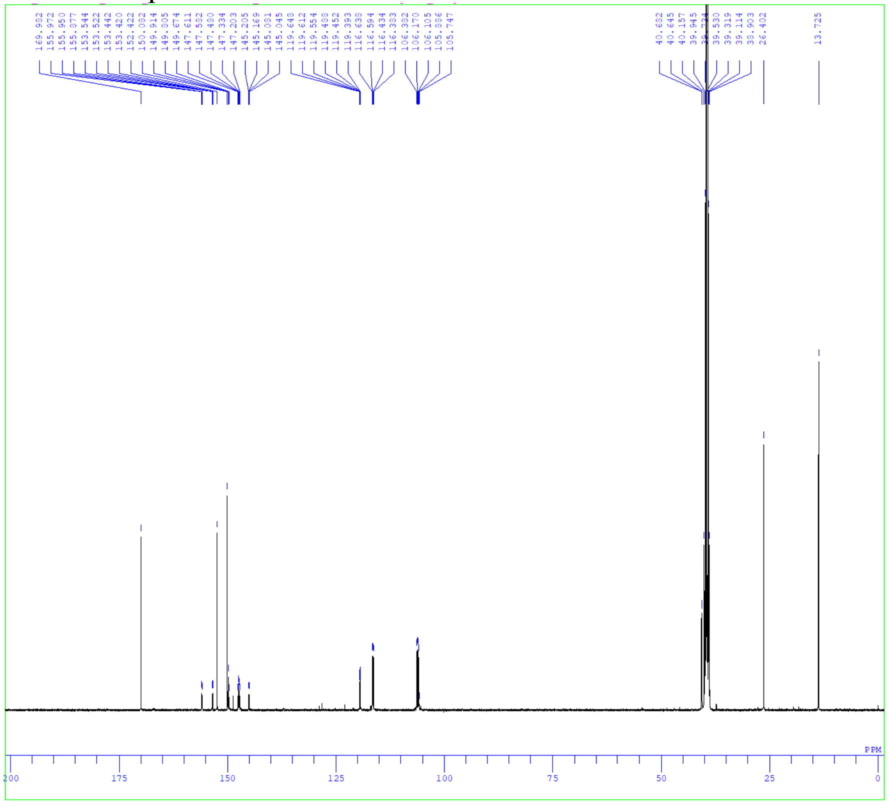

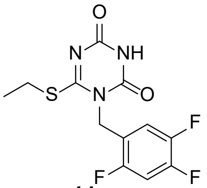

11

^{1}H NMR spectra of **12** in $CDCl_3$

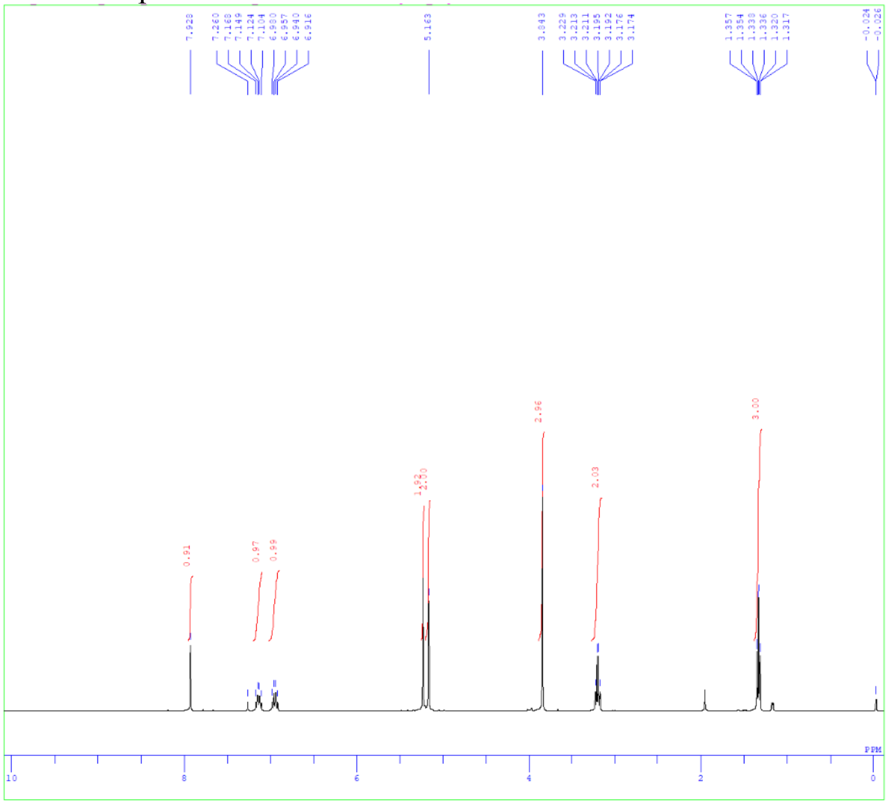

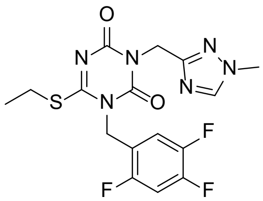

12

^{13}C NMR spectra of **12** in $CDCl_3$

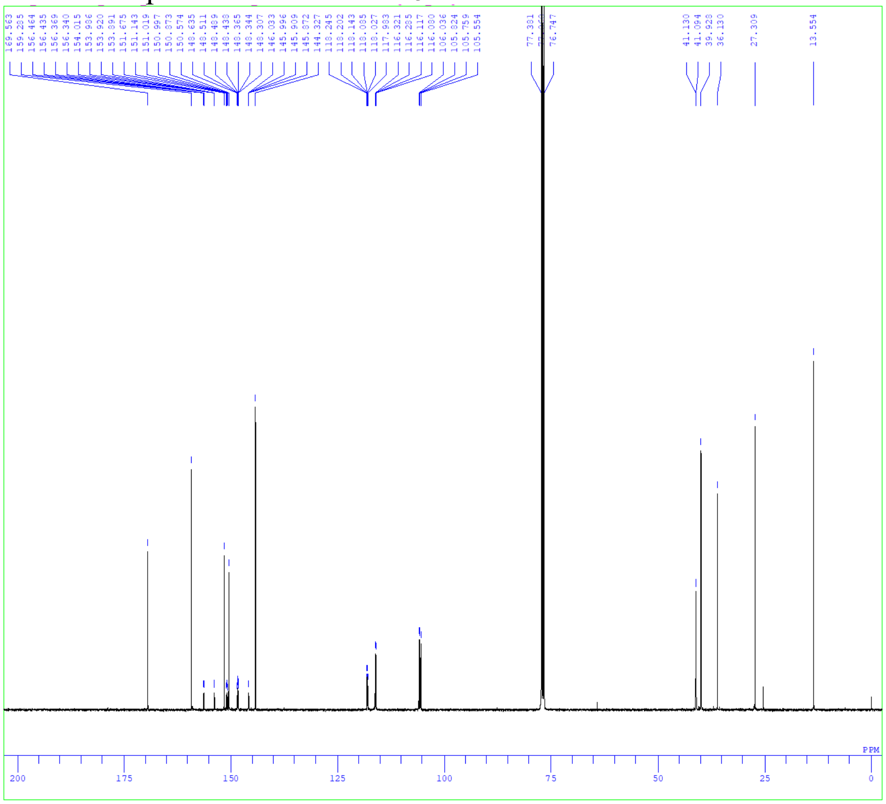

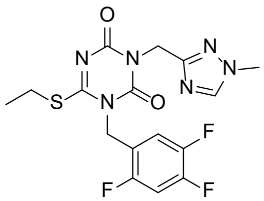

12

^{1}H NMR spectra of **3** in DMSO-d_6 with DCl in D_2O

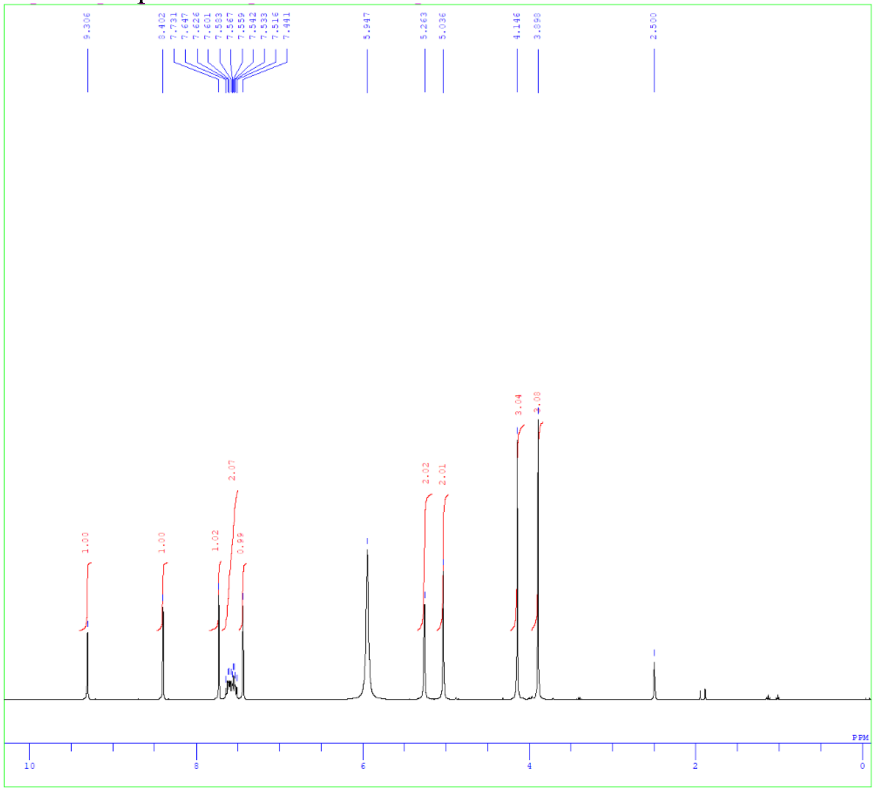

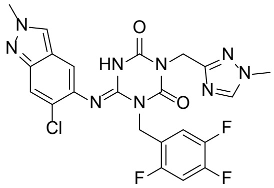

3

^{13}C NMR spectra of **3** in DMSO-d_6 with DCl in D_2O

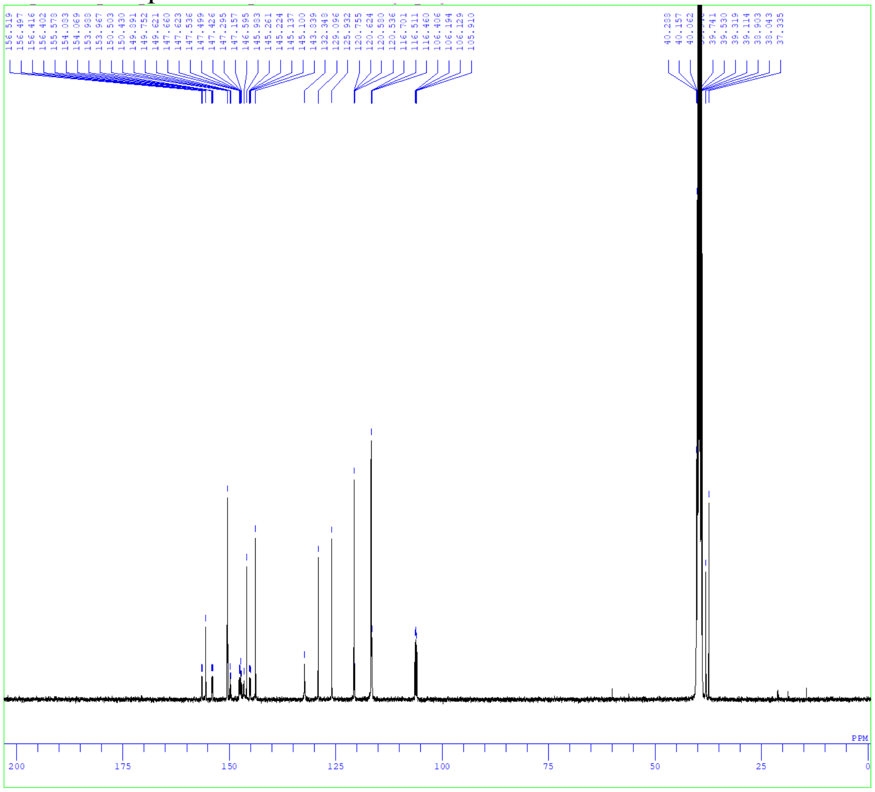

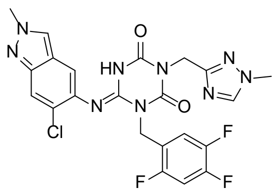

3

^{1}H NMR spectra of compound **3 (S-217622)** fumaric acid in pyridine-*d*$_5$

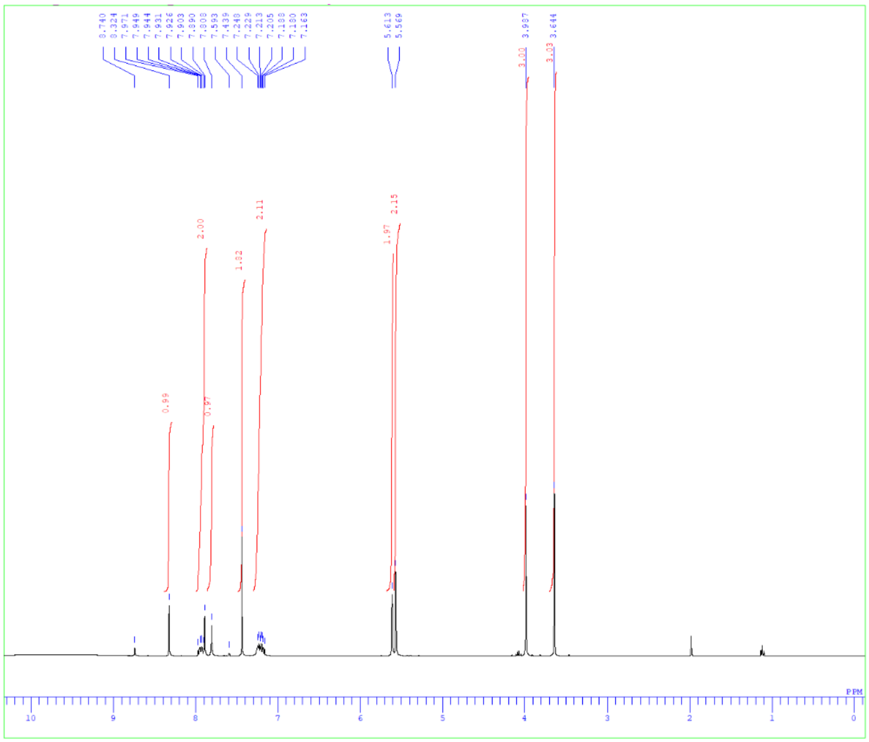

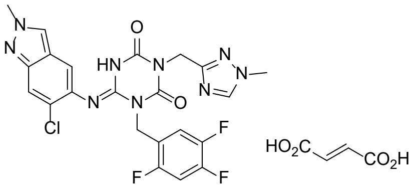

S-217622 fumalic acid

^{1}H NMR spectra of **13** in DMSO-d_6 with DCl in D_2O

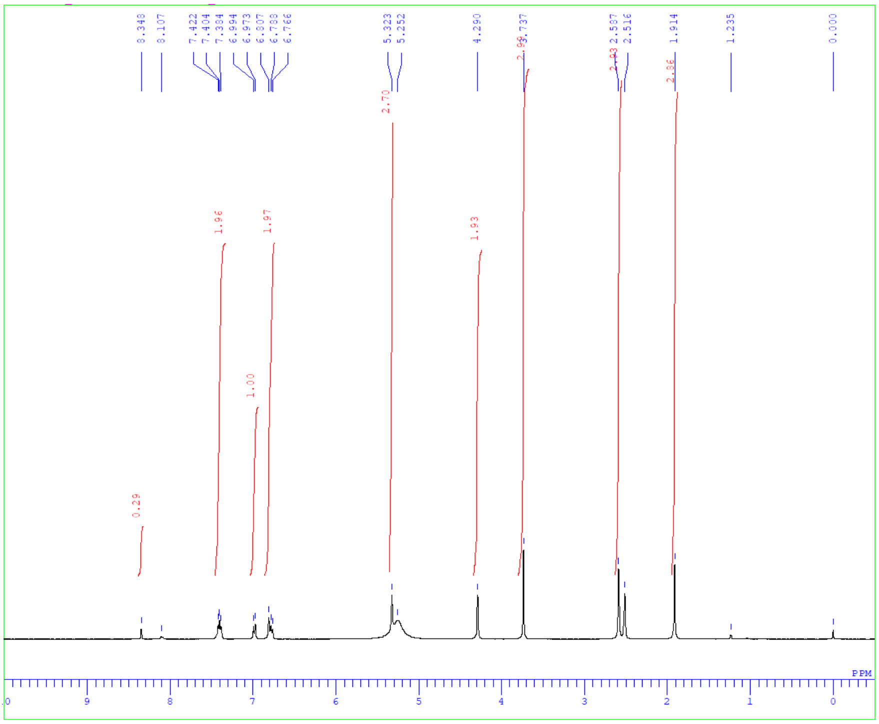

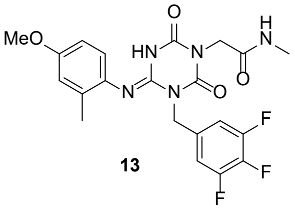

13

^{13}C NMR spectra of **13** in DMSO-d_6 with DCl in D_2O

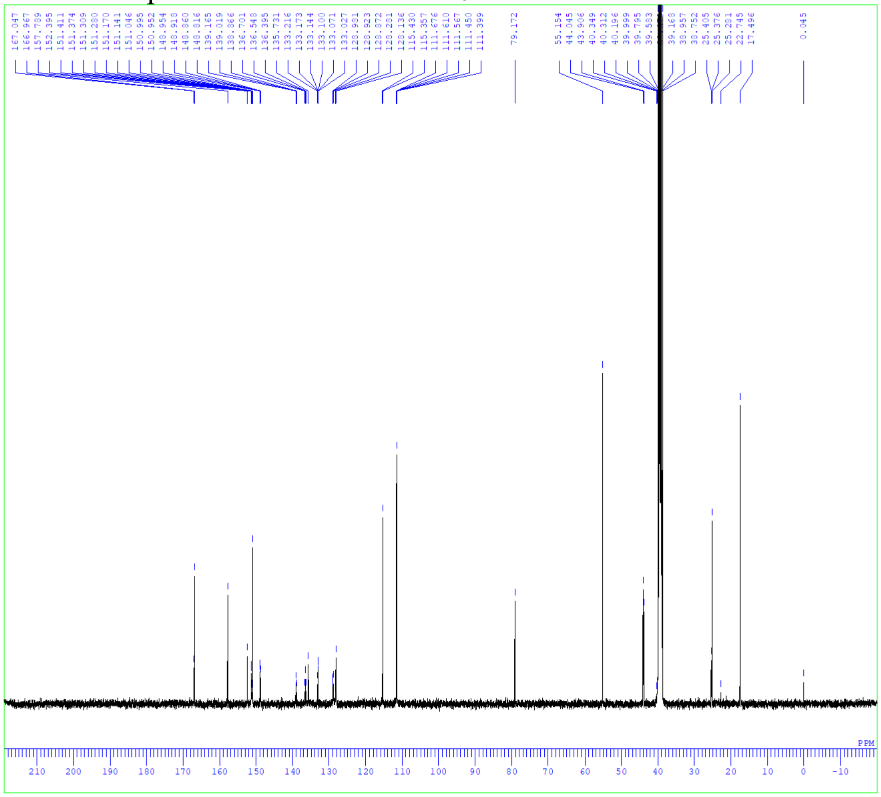

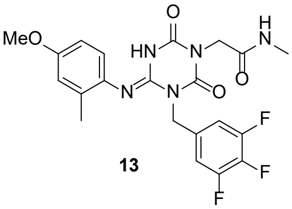

13

^1H NMR spectra of **14** in DMSO-d_6 with DCl in D_2O

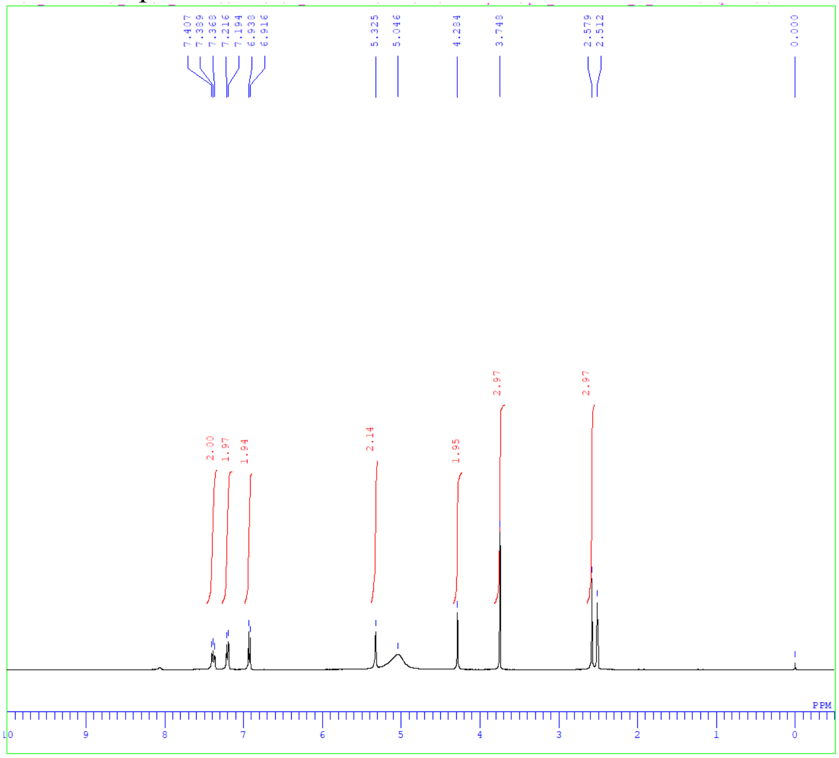

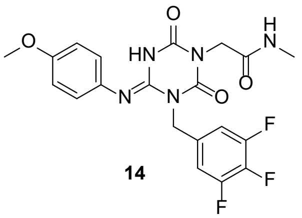

^{13}C NMR spectra of **14** in DMSO-d_6 with DCl in D_2O

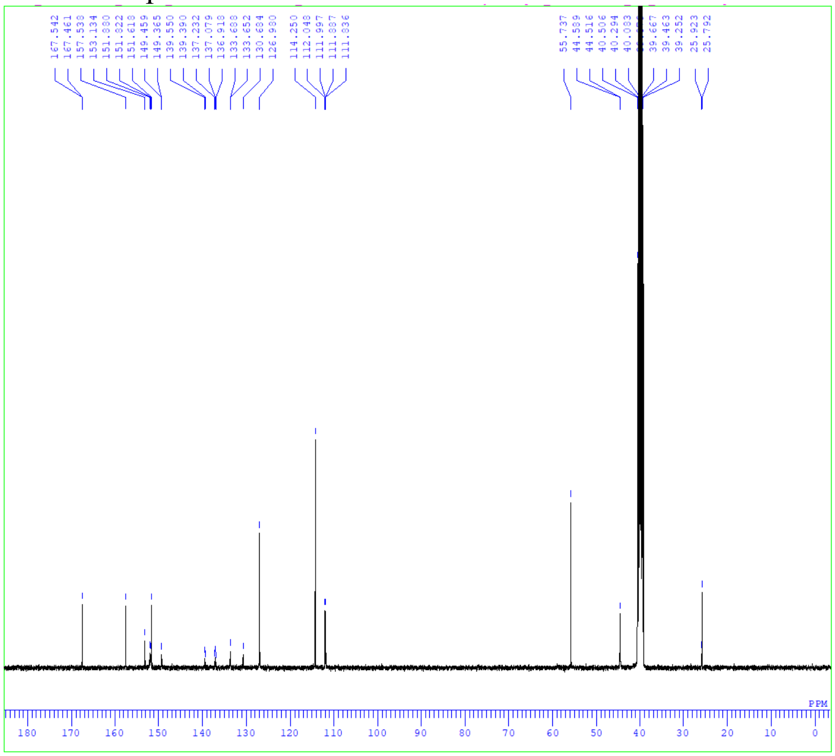

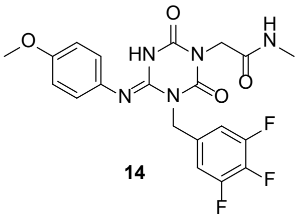

^{1}H NMR spectra of **15** in $CDCl_3$

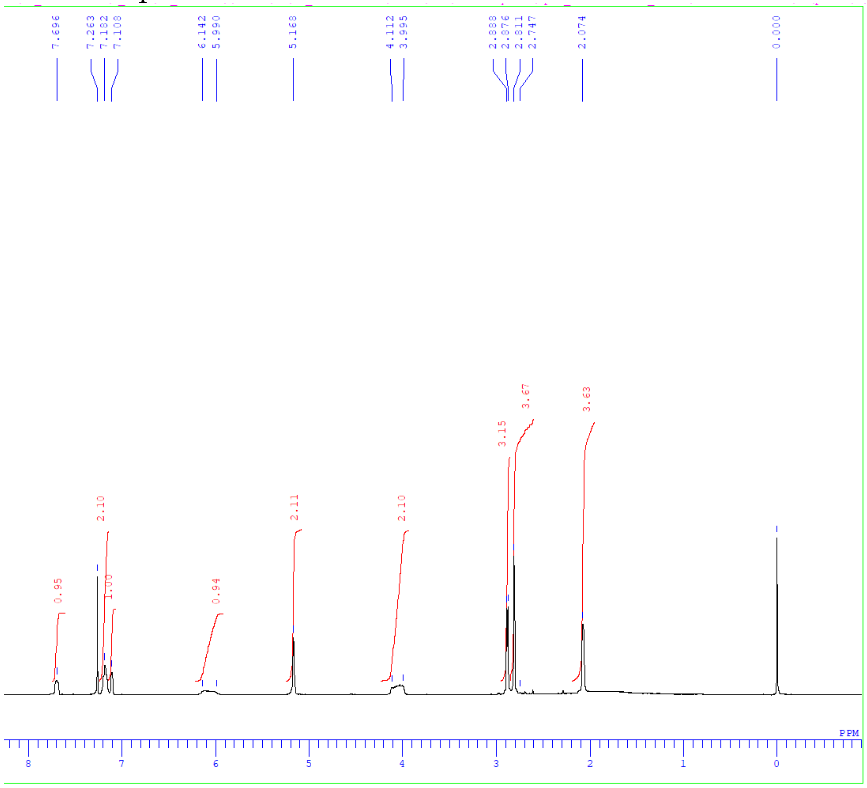

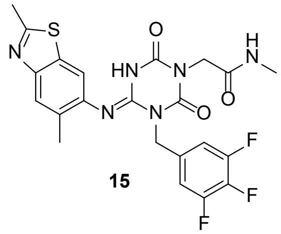

^{13}C NMR spectra of **15** in $CDCl_3$

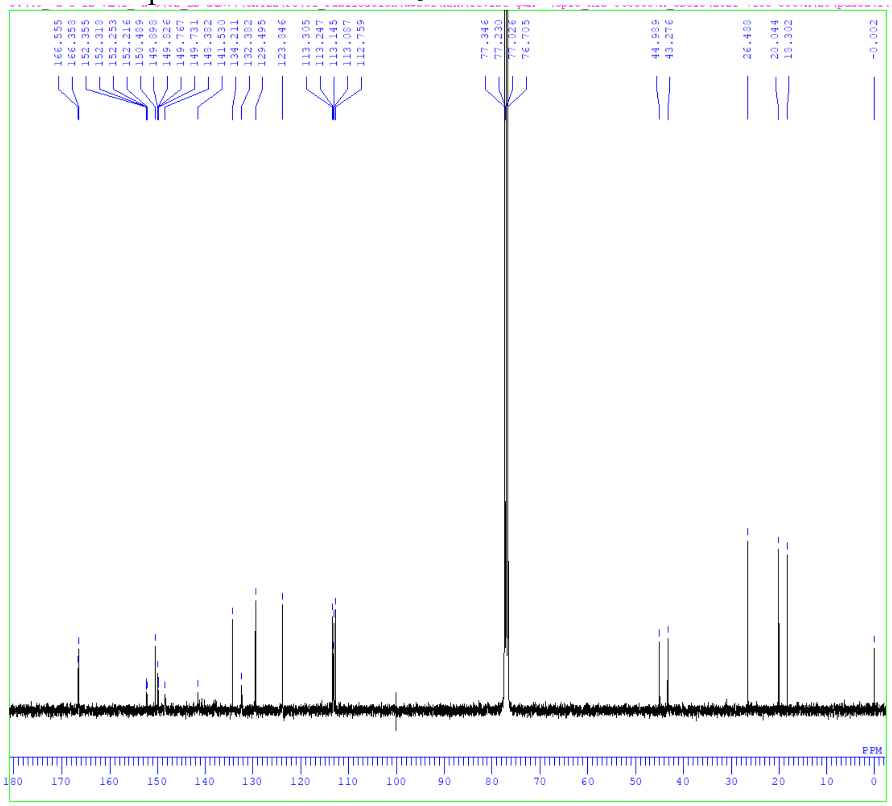

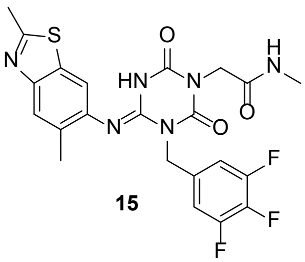

^{1}H NMR spectra of **16** in DMSO-d_6 with DCl in D_2O

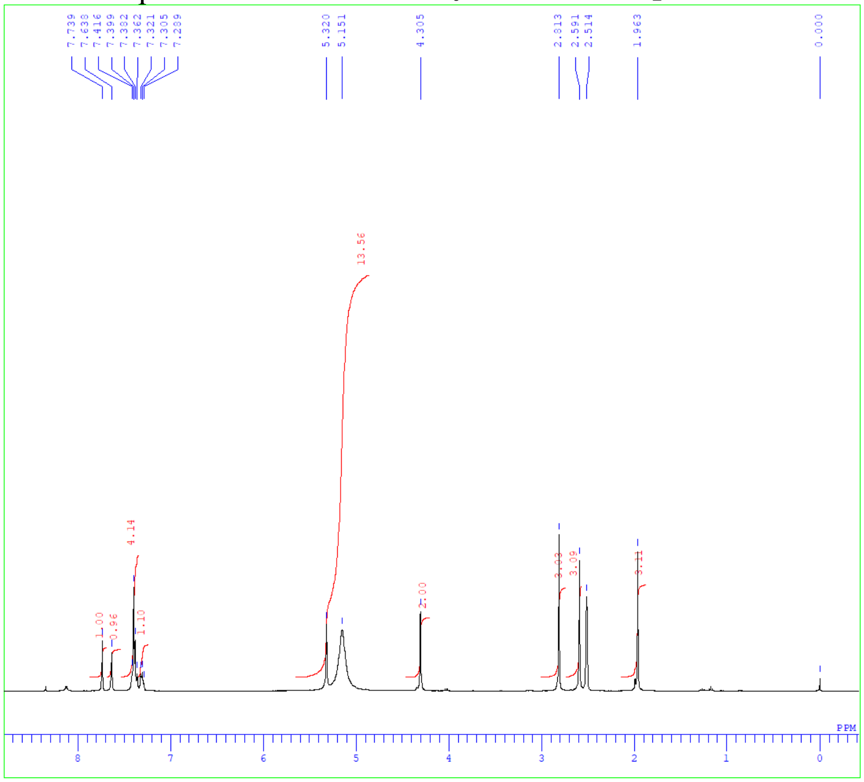

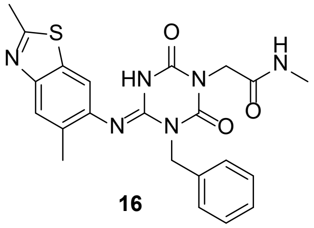

16

^{13}C NMR spectra of **16** in DMSO-d_6 with DCl in D_2O

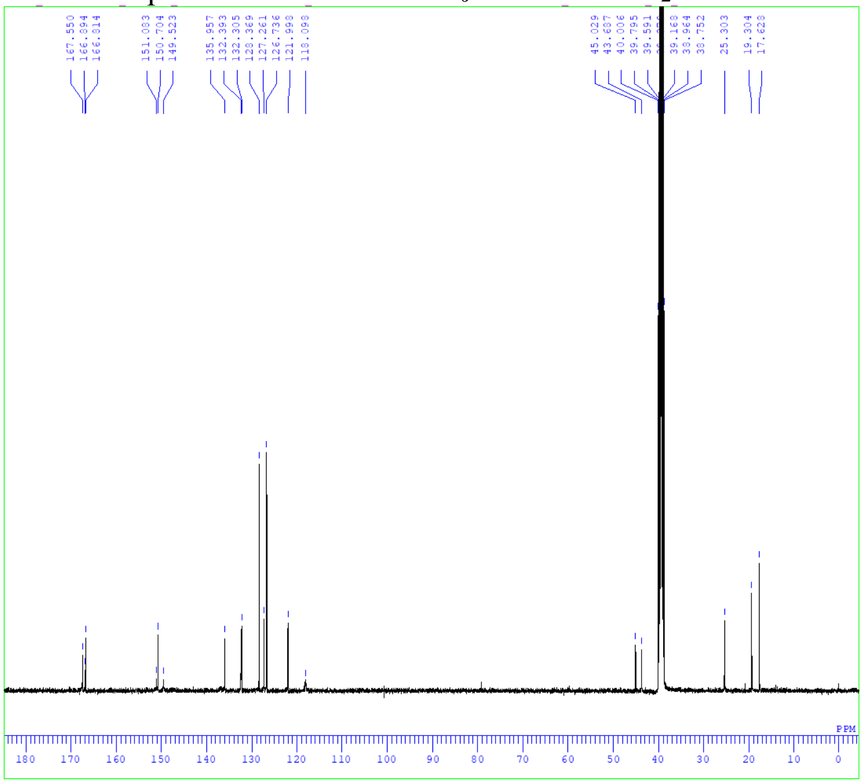

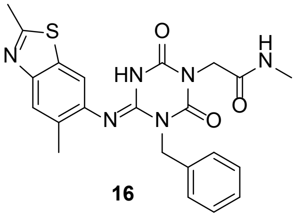

16

^{1}H NMR spectra of **17** in DMSO-d_6 with DCl in D_2O

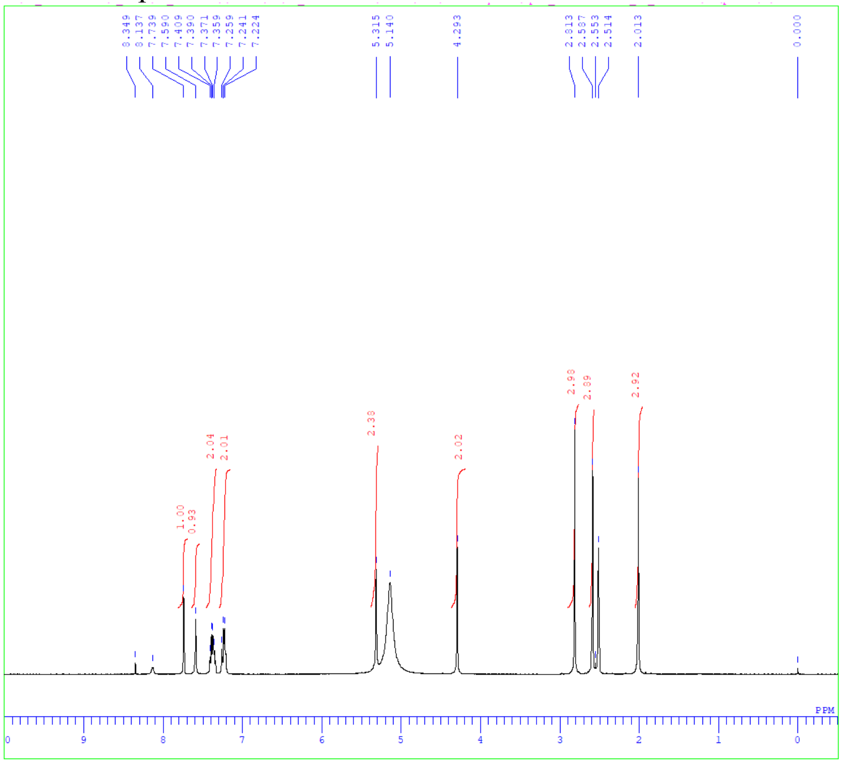

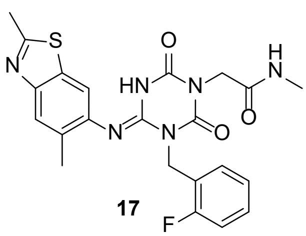

17

^{13}C NMR spectra of **17** in DMSO-d_6 with DCl in D_2O

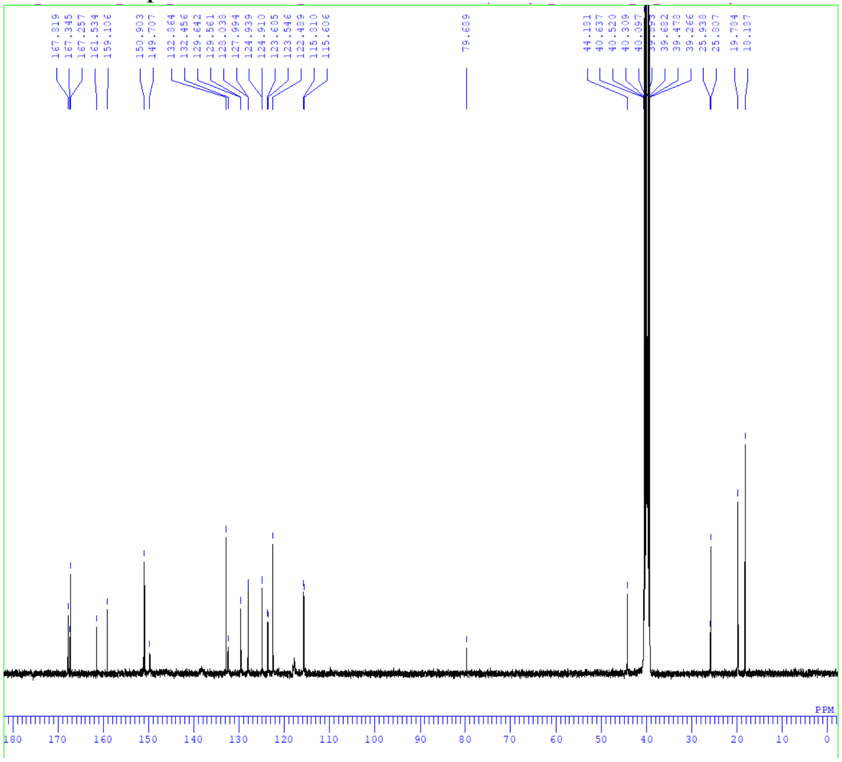

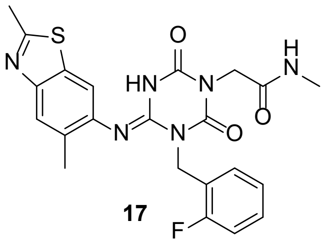

17

^{1}H NMR spectra of **18** in DMSO-d_6 with DCl in D_2O

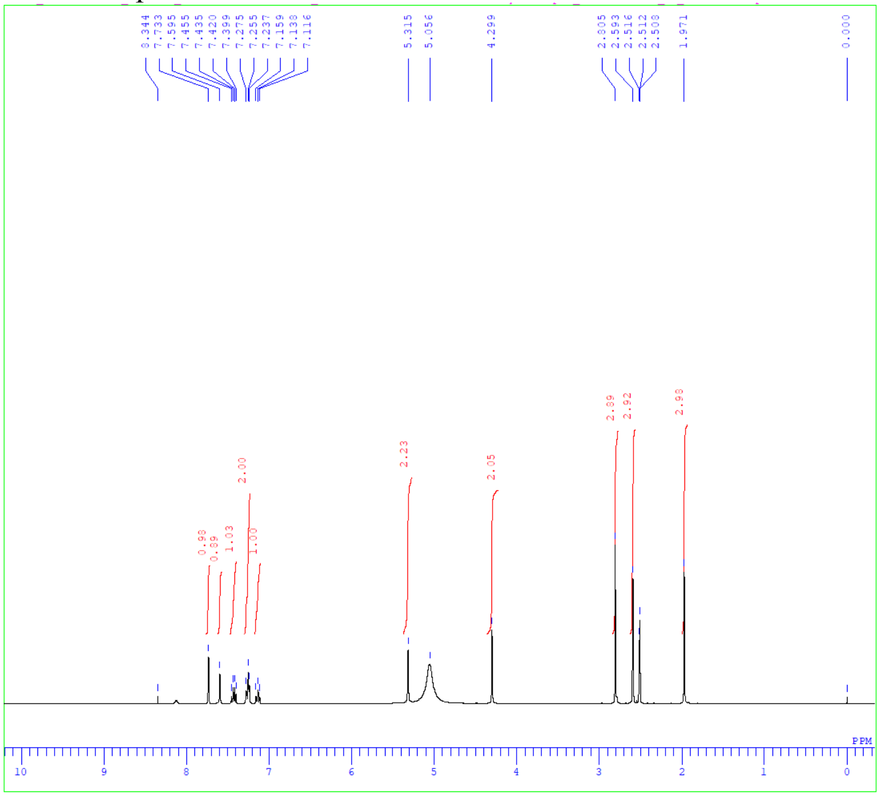

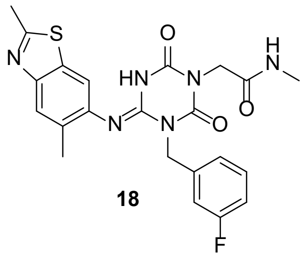

18

^{13}C NMR spectra of **18** in DMSO-d_6 with DCl in D_2O

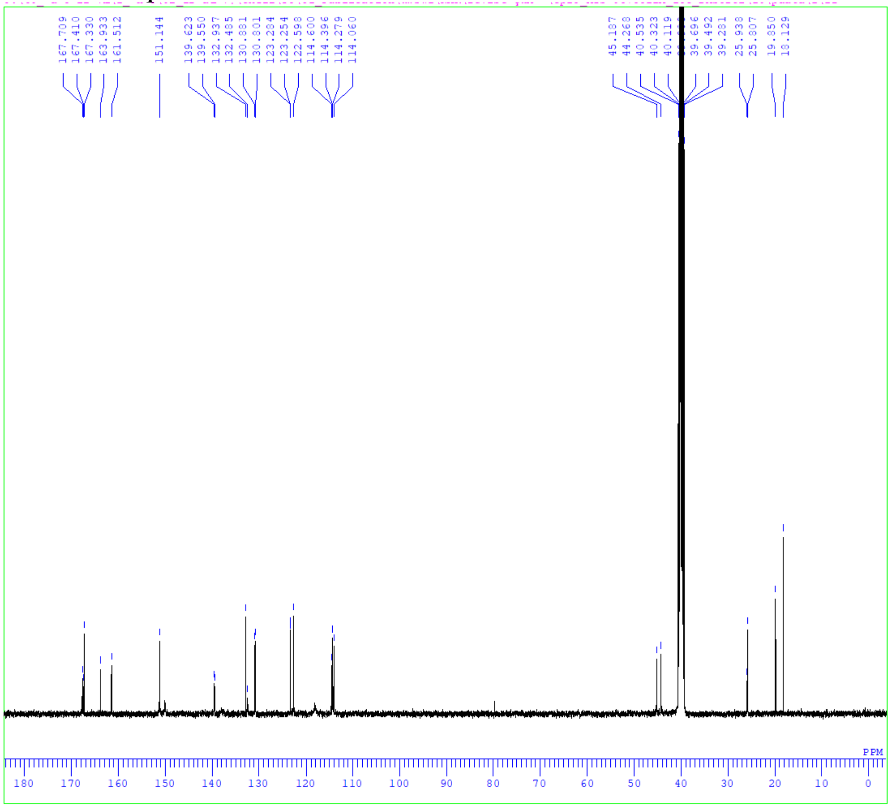

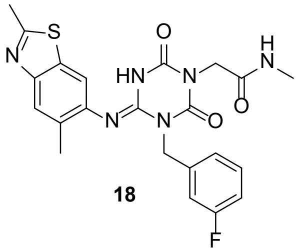

18

^{1}H NMR spectra of **19** in DMSO-d_6 with DCl in D_2O

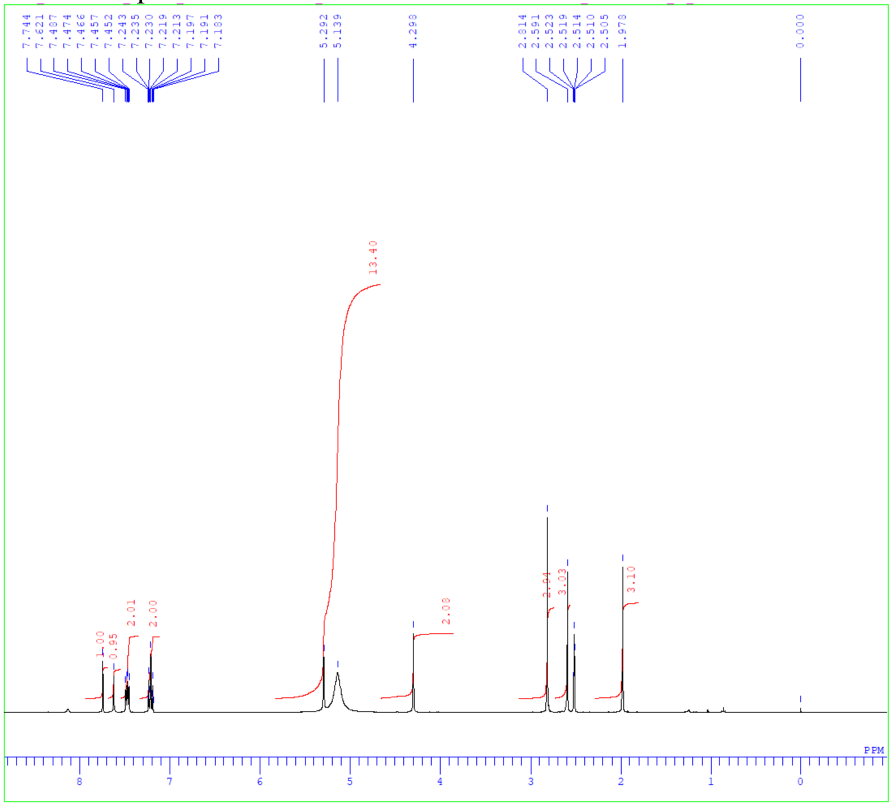

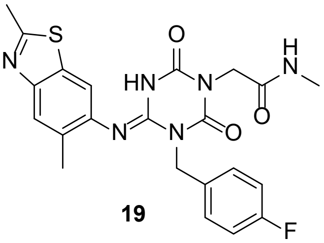

19

^{13}C NMR spectra of **19** in DMSO-d_6 with DCl in D_2O

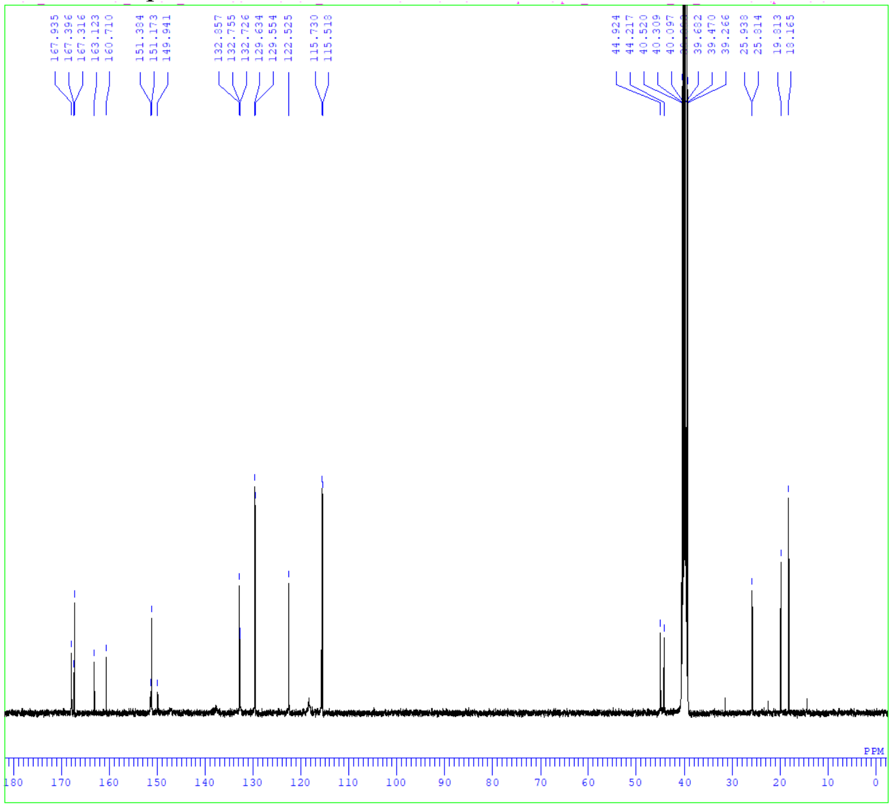

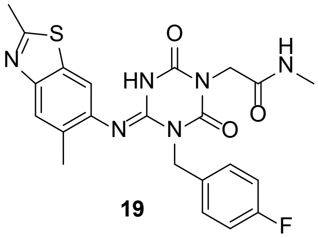

19

^{1}H NMR spectra of **20** in DMSO-d_6 with DCl in D_2O

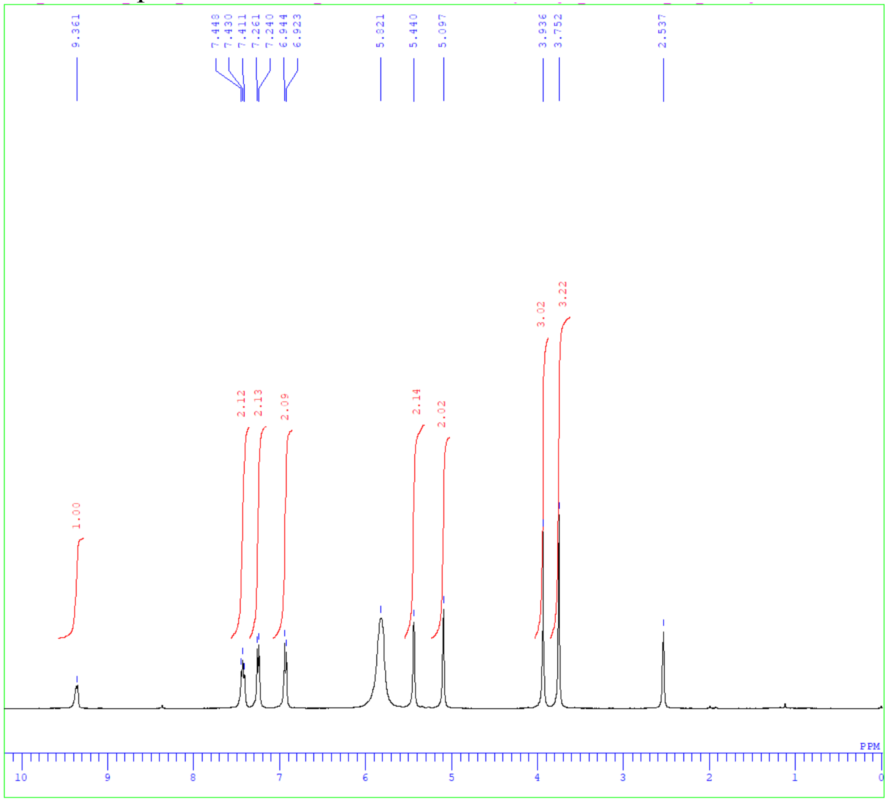

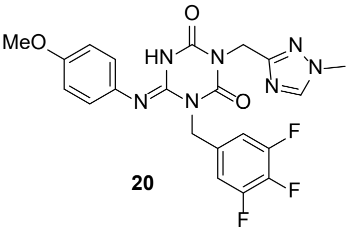

20

^{13}C NMR spectra of **20** in DMSO-d_6 with DCl in D_2O

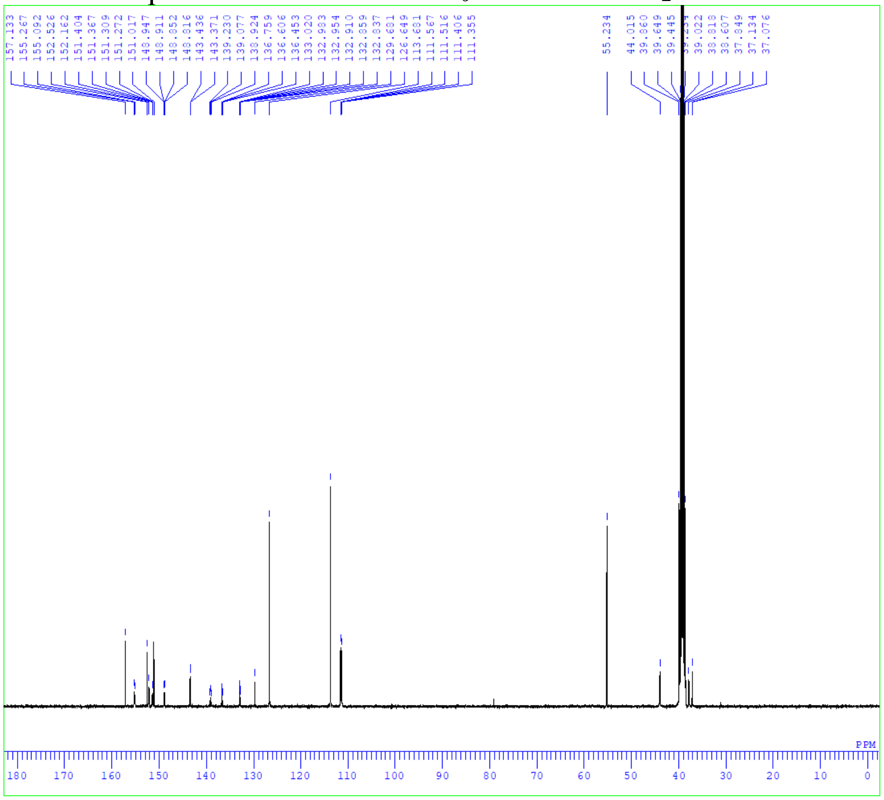

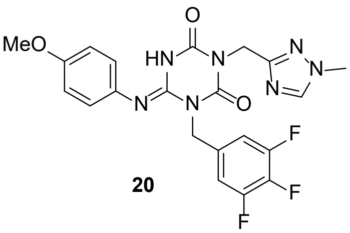

20

^{1}H NMR spectra of **21** in DMSO-d_6 with DCl in D_2O

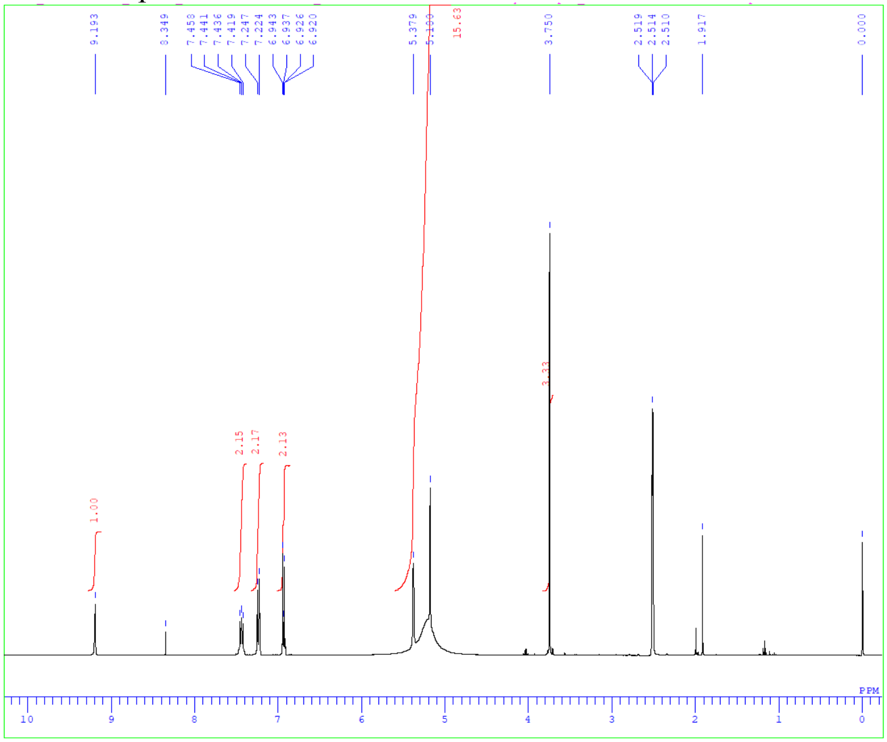

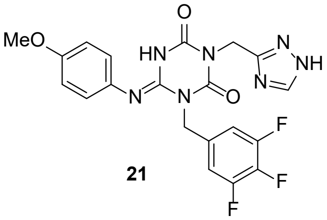

21

^{13}C NMR spectra of **21** in DMSO-d_6 with DCl in D_2O

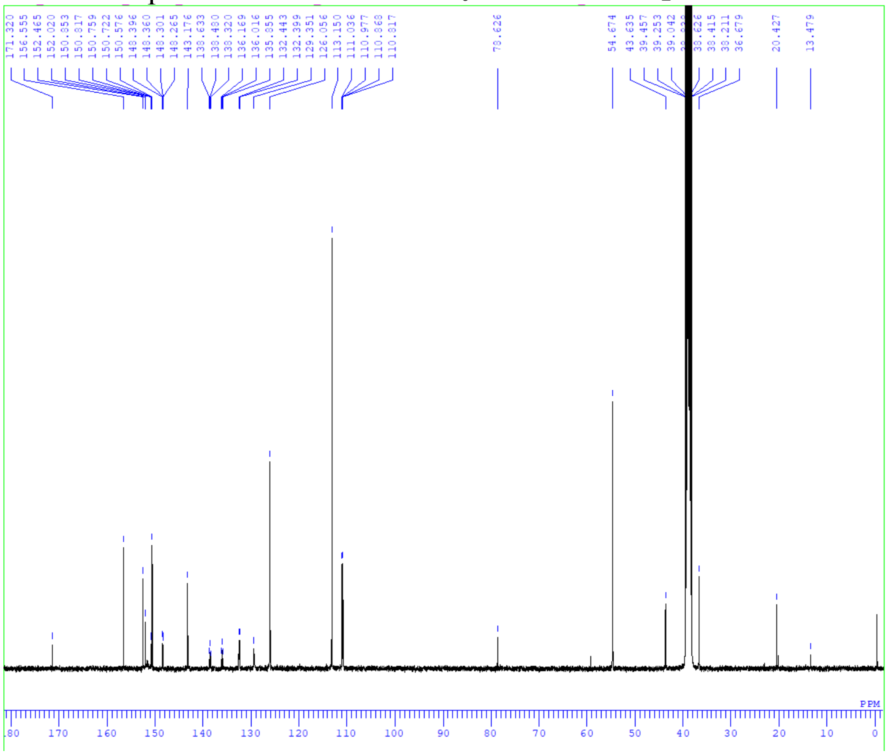

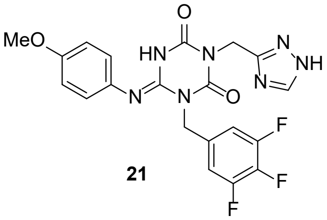

21

HPLC traces of compound 1-3

Procedure

Analytical liquid chromatography/mass spectroscopy (LC/MS) was performed on a Shimadzu Shim-pack XR-ODS (C18, 2.2 µm, 3.0 × 50 mm, linear gradient from 10% to 100% B over 3 min, then 100% B for 1 min [A = water + 0.1% formic acid, B = MeCN + 0.1% formic acid], flow rate: 1.6 mL/min) using a Shimadzu UFLC system equipped with a LCMS-2020 mass spectrometer, LC-20AD binary gradient module, SPD-M20A photodiode array detector (detection at 254 nm), and SIL-20AC sample manager.

Compound **1**

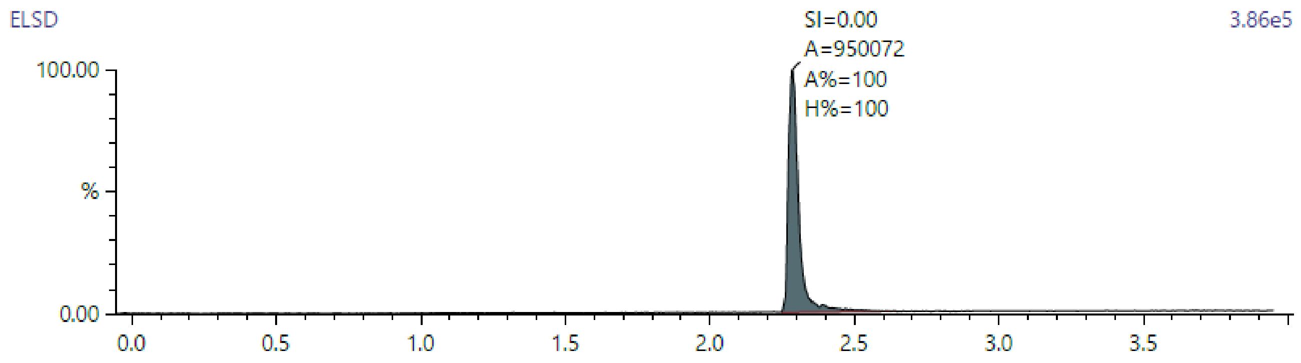

Pk#	RT	Area	Area %	Height	Height %
6	2.286	950,072	100.000	376,089	100.000
**Totals:**		**950,072**	**100.000**	**376,089**	**100.000**

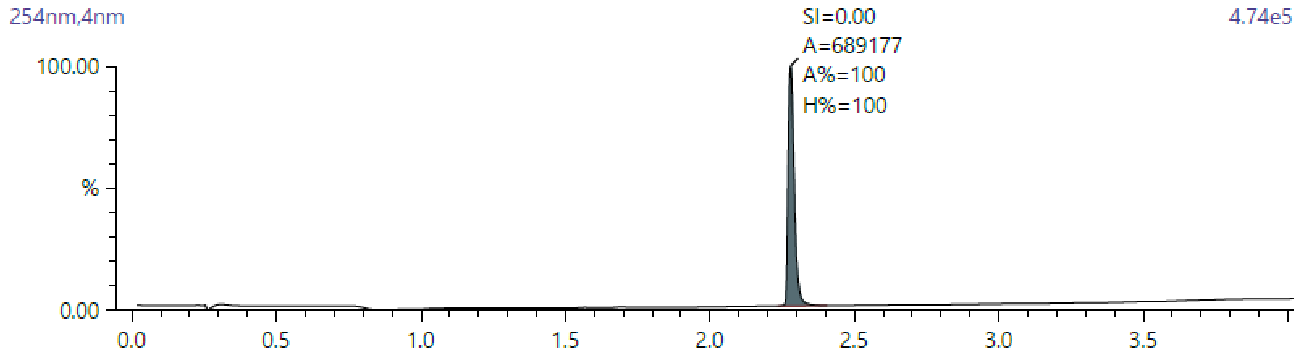

Pk#	RT	Area	Area %	Height	Height %
6	2.279	689,177	100.000	474,219	100.000
**Totals:**		**689,177**	**100.000**	**474,219**	**100.000**

Compound **2**

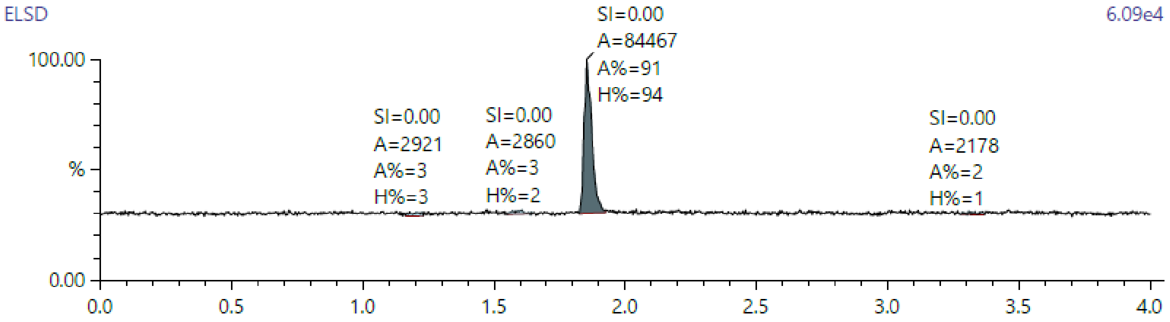

Pk#	RT	Area	Area %	Height	Height %
1	1.180	2,921	3.161	1,204	2.665
2	1.607	2,860	3.094	1,126	2.492
3	1.854	84,467	91.389	42,478	94.042
4	3.297	2,178	2.356	362	0.801
**Totals:**		**92,426**	**100.000**	**45,169**	**100.000**

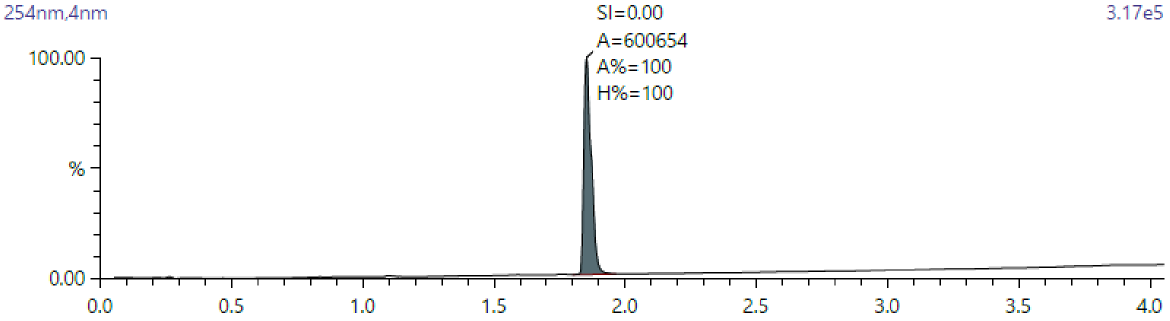

Pk#	RT	Area	Area %	Height	Height %
3	1.853	600,654	100.000	312,342	100.000
**Totals:**		**600,654**	**100.000**	**312,342**	**100.000**

Compound **3** (free form)

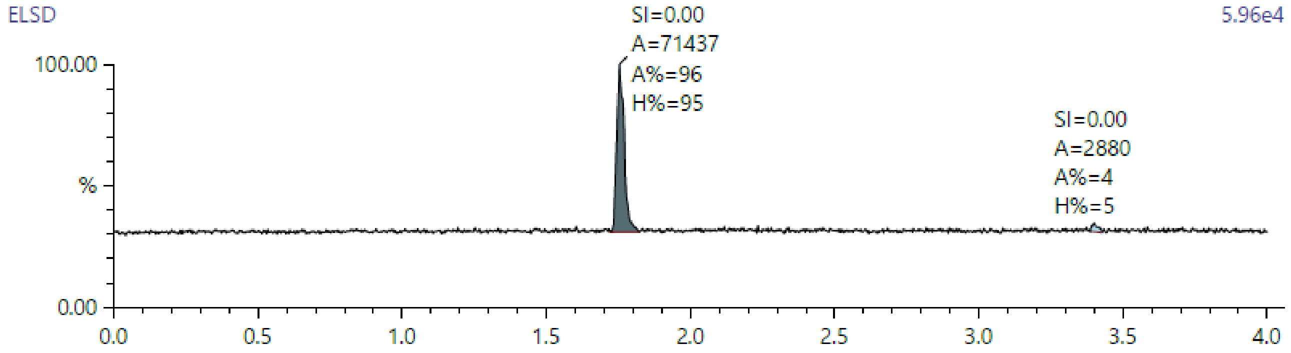

Pk#	RT	Area	Area %	Height	Height %
1	1.754	71,437	96.124	41,034	95.111
2	3.399	2,880	3.876	2,109	4.889
**Totals:**		**74,317**	**100.000**	**43,144**	**100.000**

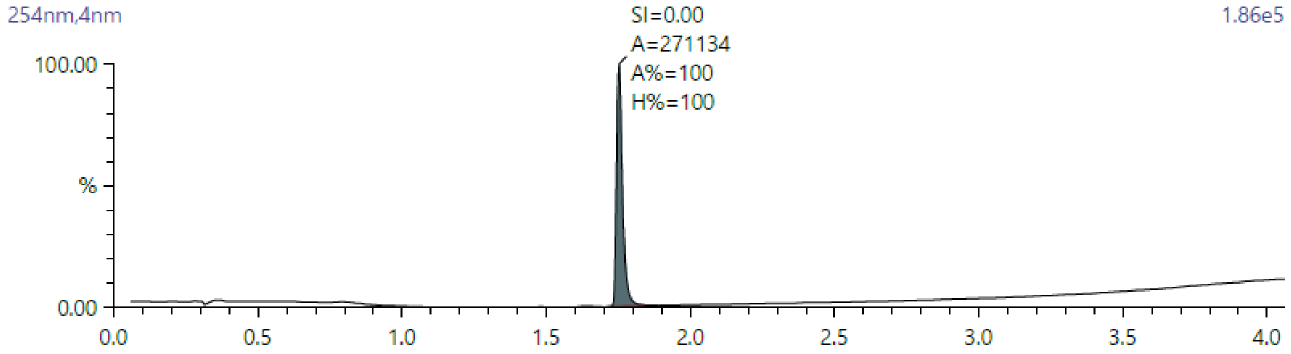

Pk#	RT	Area	Area %	Height	Height %
1	1.752	271,134	100.000	189,771	100.000
**Totals:**		**271,134**	**100.000**	**189,771**	**100.000**

HPLC chromatogram of S-217622 fumaric acid

Analytical liquid chromatography of S-217622 fumaric acid was performed on a ACQUITY UPLC BEH C18 Column (1.7 µm, 2.1 mm I.D.x100 mm, gradient from 10% to 100% B [A = 10 mmol/L $HCOONH_4$ /H_2O, B = MeCN], flow rate: 0.3 mL/min) using a Shimadzu Nexera system equipped with LC-20AD binary gradient module and SPD-20AV detector (detection at 255 nm).

Time-program for gradient elution

Time (min)	Mobile Phase B (%)
0	10
3	30
20	30
26	90
32	90
32.01	10
42	Stop

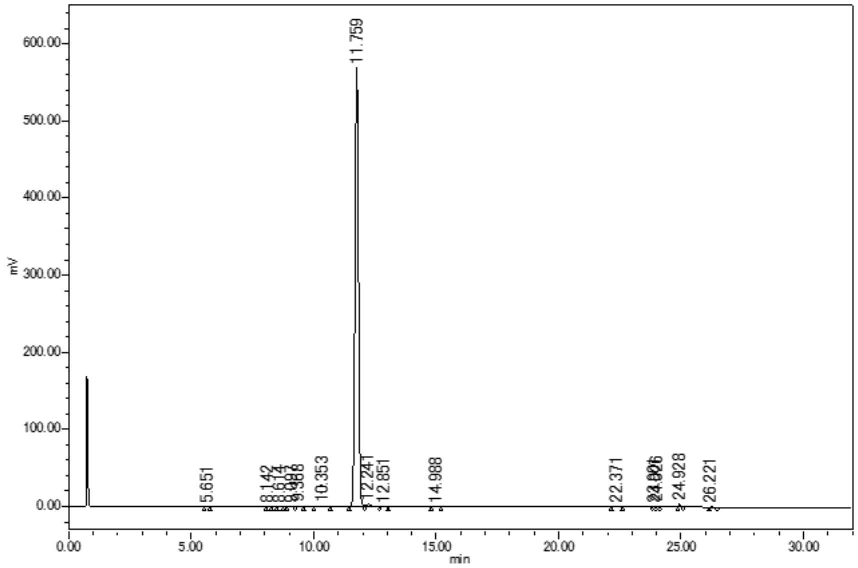

Retention time (min)	Peak area (µV·sec)	Peak height (µV)	% area
5.7	1179	320	0.02
8.1	1119	212	0.02
8.6	1316	219	0.02
9.1	1095	132	0.02
9.4	6948	987	0.12
10.4	14968	881	0.26
11.8	5559146	569193	98.21
12.2	45115	3668	0.80
12.9	1613	151	0.03
15.0	1242	102	0.02
22.4	4185	364	0.07
23.9	1721	436	0.03
24.0	4907	1408	0.09
24.9	13517	4116	0.24
26.2	2513	410	0.04

Experimental Procedures for *in vitro* safety.

Human ether-a-go-go-related gene inhibition assay

To evaluate an electrocardiogram QT interval prolongation of S-217622, effects on delayed rectifier K+ current (IKr) were evaluated using Chinese hamster ovary (CHO) cells expressing the human ether-a-go-go-related gene channel. The study was conducted in compliance with good laboratory practice regulations.

CHO cells were retained at a membrane potential of −80 mV with a whole-cell patch-clamp system (EPC-10 amplifier/PatchMaster v2.8 software, HEKA Co., Ltd.), and IKr was elicited via repolarization pulse at −40 mV for 2 sec after a depolarization pulse at +20 mV for 1 sec. S-217622 fumaric acid was dissolved in DMSO and diluted 200-fold with external solution to prepare an objective concentration. The S-217622 concentration levels were 10, 30 and 100 μM. E-4031 at 0.1 μM and the vehicle (0.5% DMSO) were applied as positive and negative controls, respectively.

From the recorded IKr, an absolute value of the tail peak current was measured based on the current value at the resting membrane potential using the whole-cell patch-clamp method. The percentages of the preapplication values in the test substance and control groups were compensated by the mean value of the percentage of the preapplication value in the negative-control group, and the compensated suppression rates were calculated.

Patch-clamp solutions were as follows. Internal solution: KCL: 130mmol/L, $MgCl_2$: 1 mmol/L, MgATP: 5 mmol/L, EGTA: 5 mmol/L, HEPES (4-(2-hydroxyethyl)-1-piperazineethanesulfonic acid): 10 mmol/L, pH=7.2. External solution: NaCl: 137 mmol/L, KCl: 4 mmol/L, $CaCl_2$: 1.8 mmol/L, $MgCl_2$: 1 mmol/L, glucose: 10 mmol/L, HEPES: 10 mmol/L, pH=7.4.

***In vitro* micronucleus test.** To evaluate the clastogenic potential of S-217622, an *in vitro* micronucleus test was conducted using TK6 cells (human lymphoblast-derived) in a short-term treatment with and without a metabolic activation system (S9 mix) and continuous treatment without S9 mix. S9 mix containing 9,000 g of liver supernatant fraction was prepared from Sprague-Dawley rats treated with phenobarbital and 5,6-benzofravone. The study was conducted in compliance with good laboratory practice regulations.

A TK6 cell suspension was used in the absence of metabolic activation in the 3-h and 24-h treatment groups or in the presence of metabolic activation in the 3-h treatment group. Suspensions were mixed with S-217622 fumaric acid in DMSO solution with S9 mix in the presence of metabolic activation in the 4-h

treatment group, then incubated at 37°C. The negative-control (DMSO) and positive-control (mitomycin C, cyclophosphamide monohydrate, or colchicine) substances were prepared concurrently. After the end of the short-term treatment, cells were washed and then incubated in fresh culture medium for 21 h. After incubation, the cells were counted to evaluate cytotoxicity, and the *in vitro* micronucleus test was conducted with S-217622 fumaric acid at doses of 150–250 μg/mL for short-term treatment and 75–125 μg/mL for continuous treatment based on cytotoxicity. The nucleic acid was stained with acridine orange, and the micronuclear frequency of the specimens was observed under a fluorescence microscope. The test was considered positive when a significant and dose-dependent increase was noted in the number of cells with micronuclei in the test-substance groups compared with the negative-control group under any treatment condition.

Ames test. To evaluate the mutagenic potential of S-217622, a bacterial reverse-mutation test was conducted via the preincubation method using five bacterial strains, including *Salmonella typhimurium* (TA98, TA100, TA1535, and TA1537) and *Escherichia coli* (WP2*uvrA*) in the presence or absence of a metabolic activation system (S9 mix). S9 mix containing 9,000 g of liver supernatant fraction was prepared from Sprague-Dawley rats treated with phenobarbital and 5,6-benzofravone. The study was conducted in compliance with good laboratory practice regulations.

S-217622 fumaric acid (DMSO solution) was mixed with S9 mix in the presence of metabolic activation or phosphate buffer in the absence of metabolic activation and 0.1 mL of test strain suspension (1×10^9 cells/mL or greater) and incubated at 37°C for 20 min. Then, the mixture with a layer of soft agar containing histidine and biotin or tryptophan was overlaid on minimal glucose agar plates. The negative-control (DMSO) and positive-control (4-nitroquinoline 1-oxide, sodium azide, 9-aminoacridine hydrochloride monohydrate, or 2-aminoanthracene) substances were prepared concurrently. The mutation test was conducted with S-217622 fumaric acid at 156–5000 μg/plate in TA98, TA100, TA1535, and WP2*uvrA* and at 39.1–5000 μg/plate in TA1537. After incubation at 37°C for 48 h, the revertant colonies were counted and evaluated by comparing them with the negative-control group. The test was considered positive when the number of revertant colonies was concentration-dependently increased and twofold or greater increased over the number of colonies of the negative-control group.

***In vitro* 3T3.** To evaluate the phototoxicity potential of S-217622, an *in vitro* phototoxicity study was conducted with cultured mammalian cells. The study was conducted in compliance with good laboratory

practice regulations. A fibroblastic cell line derived from BALB/c mice (BALB/3T3 cells) was cultured in 96-well plates and treated with S-217622 fumaric acid for 1 h followed by UV-A (5 J/cm^2) and UV-B (68.7 mJ/cm^2) irradiation. For the comparator, no irradiation was conducted. The phototoxicity test was performed at 0.781–100 µg/mL. A vehicle (DMSO)-treated group and a chlorpromazine hydrochloride-treated group were set as the negative and positive controls, respectively. Cell viability was determined by neutral-red extraction from cells (measurement of absorbance at 540 nm). When the IC_{50} could be determined for both the irradiated and nonirradiated plates, the result was determined from the PIF. When the IC_{50} could not be determined for either the irradiated or nonirradiated plates, the result was determined from the MPE. The judgment criteria are shown below.

No phototoxicity: PIF < 5 or MPE < 0.15
Phototoxicity: $5 \leq$ PIF or $0.15 \leq$ MPE
