# Supplementary material for: Discovery of S-217622, a Noncovalent Oral SARS-CoV-2 3CL Protease Inhibitor Clinical Candidate for Treating COVID-19
Source: J Med Chem. 2022 Mar 30;65(9):6499–512. doi: 10.1021/acs.jmedchem.2c00117 (PMC8982737; doi:10.1021/acs.jmedchem.2c00117)
Supplement: Supplementary file 4 — jm2c00117_si_004.pdf [file jm2c00117_si_004.pdf]

Full wwPDB X-ray Structure Validation Report (i)

Nov 8, 2021 – 04:38 PM JST

PDB ID	:	7VTH
Title	:	The crystal structure of SARS-CoV-2 3CL protease in complex with compound 1
Deposited on	:	2021-10-29
Resolution	:	2.00 Å(reported)

CONFIDENTIAL VALIDATION REPORT

This is a Full wwPDB X-ray Structure Validation Report.

This report is produced by the wwPDB biocuration pipeline after annotation of the structure.

We welcome your comments at *validation@mail.wwpdb.org*
A user guide is available at
https://www.wwpdb.org/validation/2017/XrayValidationReportHelp
with specific help available everywhere you see the (i) symbol.

The following versions of software and data (see references (i)) were used in the production of this report:

MolProbity	:	4.02b-467
Mogul	:	1.8.5 (274361), CSD as541be (2020)
Xtriage (Phenix)	:	1.13
EDS	:	2.23.2
buster-report	:	1.1.7 (2018)
Percentile statistics	:	20191225.v01 (using entries in the PDB archive December 25th 2019)
Refmac	:	5.8.0158
CCP4	:	7.0.044 (Gargrove)
Ideal geometry (proteins)	:	Engh & Huber (2001)
Ideal geometry (DNA, RNA)	:	Parkinson et al. (1996)
Validation Pipeline (wwPDB-VP)	:	2.23.2

1 Overall quality at a glance (i)

The following experimental techniques were used to determine the structure:
X-RAY DIFFRACTION

The reported resolution of this entry is 2.00 Å.

Percentile scores (ranging between 0-100) for global validation metrics of the entry are shown in the following graphic. The table shows the number of entries on which the scores are based.

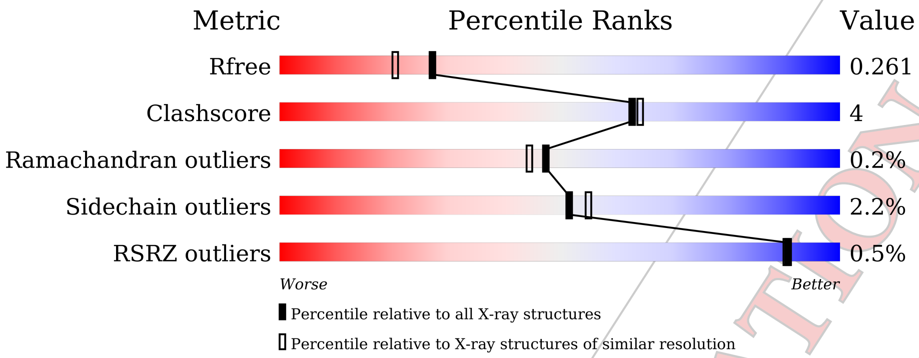

Metric	Whole archive (#Entries)	Similar resolution (#Entries, resolution range(Å))
R_{free}	130704	8085 (2.00-2.00)
Clashscore	141614	9178 (2.00-2.00)
Ramachandran outliers	138981	9054 (2.00-2.00)
Sidechain outliers	138945	9053 (2.00-2.00)
RSRZ outliers	127900	7900 (2.00-2.00)

The table below summarises the geometric issues observed across the polymeric chains and their fit to the electron density. The red, orange, yellow and green segments of the lower bar indicate the fraction of residues that contain outliers for >=3, 2, 1 and 0 types of geometric quality criteria respectively. A grey segment represents the fraction of residues that are not modelled. The numeric value for each fraction is indicated below the corresponding segment, with a dot representing fractions <=5% The upper red bar (where present) indicates the fraction of residues that have poor fit to the electron density. The numeric value is given above the bar.

Mol	Chain	Length	Quality of chain
1	A	311	% / 86% / 9% / •
1	B	311	89% / 8% / •

CONFIDENTIAL VALIDATION REPORT

2 Entry composition (i)

There are 3 unique types of molecules in this entry. The entry contains 4737 atoms, of which 0 are hydrogens and 0 are deuteriums.

In the tables below, the ZeroOcc column contains the number of atoms modelled with zero occupancy, the AltConf column contains the number of residues with at least one atom in alternate conformation and the Trace column contains the number of residues modelled with at most 2 atoms.

- Molecule 1 is a protein called 3C-like proteinase.

Mol	Chain	Residues	Atoms					ZeroOcc	AltConf	Trace
			Total	C	N	O	S			
1	A	298	2194	1397	361	415	21	5	1	0
1	B	301	2255	1435	373	423	24	0	2	0

There are 10 discrepancies between the modelled and reference sequences:

Chain	Residue	Modelled	Actual	Comment	Reference
A	0	MET	-	initiating methionine	UNP P0DTC1
A	307	LEU	-	expression tag	UNP P0DTC1
A	308	VAL	-	expression tag	UNP P0DTC1
A	309	PRO	-	expression tag	UNP P0DTC1
A	310	ARG	-	expression tag	UNP P0DTC1
B	0	MET	-	initiating methionine	UNP P0DTC1
B	307	LEU	-	expression tag	UNP P0DTC1
B	308	VAL	-	expression tag	UNP P0DTC1
B	309	PRO	-	expression tag	UNP P0DTC1
B	310	ARG	-	expression tag	UNP P0DTC1

- Molecule 2 is 2-[4-[[4-[bis(fluoranyl)methoxy]-2-methyl-phenyl]amino]-2,6-bis(oxidanylidene) -3-[[3,4,5-tris(fluoranyl)phenyl]methyl]-1,3,5-triazin-1-yl]-N-methyl-ethanamide (three-letter code: 7XB) (formula: $C_{21}H_{18}F_5N_5O_4$) (labeled as "Ligand of Interest" by depositor).

CONFIDENTIAL VALIDATION REPORT

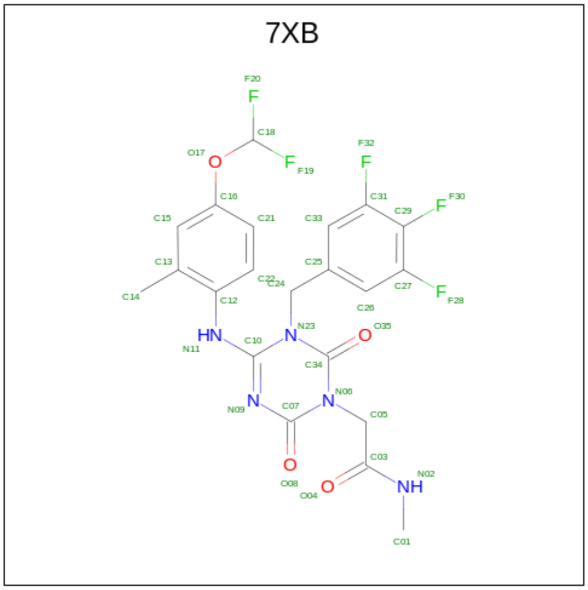

Mol	Chain	Residues	Atoms	ZeroOcc	AltConf
2	A	1	Total C F N O<br>35 21 5 5 4	0	0
2	B	1	Total C F N O<br>35 21 5 5 4	0	0

- Molecule 3 is water.

Mol	Chain	Residues	Atoms	ZeroOcc	AltConf
3	A	78	Total O<br>78 78	0	0
3	B	140	Total O<br>140 140	0	0

CONFIDENTIAL VALIDATION REPORT

3 Residue-property plots (i)

These plots are drawn for all protein, RNA, DNA and oligosaccharide chains in the entry. The first graphic for a chain summarises the proportions of the various outlier classes displayed in the second graphic. The second graphic shows the sequence view annotated by issues in geometry and electron density. Residues are color-coded according to the number of geometric quality criteria for which they contain at least one outlier: green = 0, yellow = 1, orange = 2 and red = 3 or more. A red dot above a residue indicates a poor fit to the electron density (RSRZ > 2). Stretches of 2 or more consecutive residues without any outlier are shown as a green connector. Residues present in the sample, but not in the model, are shown in grey.

- Molecule 1: 3C-like proteinase

Chain A:

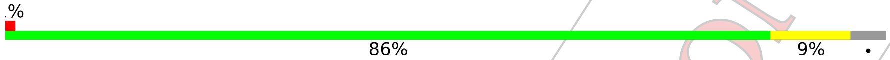

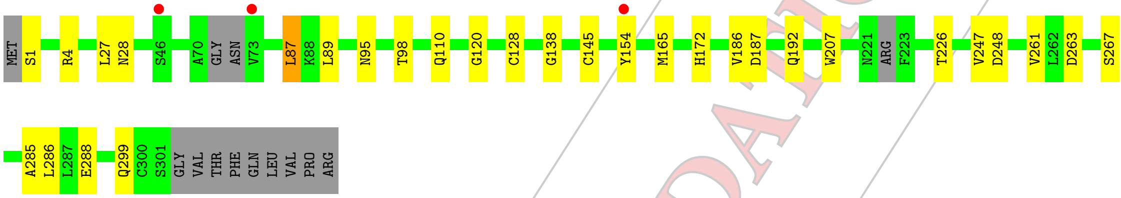

- Molecule 1: 3C-like proteinase

Chain B:

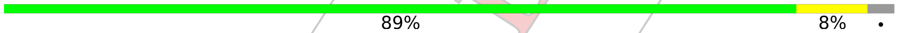

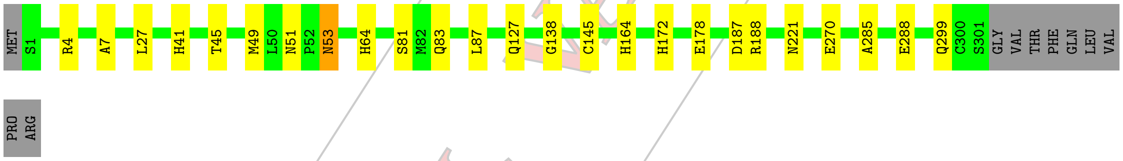

CONFIDENTIAL VALIDATION REPORT

4 Data and refinement statistics (i)

Property	Value	Source
Space group	P 1 21 1	Depositor
Cell constants<br>a, b, c, α, β, γ	44.41Å 54.27Å 114.50Å<br>90.00° 99.42° 90.00°	Depositor
Resolution (Å)	112.95 – 2.00<br>28.24 – 2.00	Depositor<br>EDS
% Data completeness<br>(in resolution range)	99.3 (112.95-2.00)<br>99.3 (28.24-2.00)	Depositor<br>EDS
R_{merge}	0.09	Depositor
R_{sym}	(Not available)	Depositor
$< I/\sigma(I) >$ [1]	2.97 (at 2.00Å)	Xtriage
Refinement program	REFMAC 5.8.0218	Depositor
R, R_{free}	0.205 , 0.257<br>0.211 , 0.261	Depositor<br>DCC
R_{free} test set	1920 reflections (5.29%)	wwPDB-VP
Wilson B-factor (Å^2)	16.5	Xtriage
Anisotropy	0.006	Xtriage
Bulk solvent k_{sol}(e/Å^3), B_{sol}(Å^2)	0.32 , 40.1	EDS
L-test for twinning[2]	$< \|L\| > = 0.45$, $< L^2 > = 0.28$	Xtriage
Estimated twinning fraction	0.030 for h,-k,-h-l	Xtriage
F_o,F_c correlation	0.93	EDS
Total number of atoms	4737	wwPDB-VP
Average B, all atoms (Å^2)	19.0	wwPDB-VP

Xtriage's analysis on translational NCS is as follows: *The analyses of the Patterson function reveals a significant off-origin peak that is 34.66 % of the origin peak, indicating pseudo-translational symmetry. The chance of finding a peak of this or larger height randomly in a structure without pseudo-translational symmetry is equal to 6.5867e-04. The detected translational NCS is most likely also responsible for the elevated intensity ratio.*

[1] Intensities estimated from amplitudes.

[2] Theoretical values of $< |L| >$, $< L^2 >$ for acentric reflections are 0.5, 0.333 respectively for untwinned datasets, and 0.375, 0.2 for perfectly twinned datasets.

CONFIDENTIAL VALIDATION REPORT

5 Model quality (i)

5.1 Standard geometry (i)

Bond lengths and bond angles in the following residue types are not validated in this section: 7XB

The Z score for a bond length (or angle) is the number of standard deviations the observed value is removed from the expected value. A bond length (or angle) with $|Z| > 5$ is considered an outlier worth inspection. RMSZ is the root-mean-square of all Z scores of the bond lengths (or angles).

Mol	Chain	Bond lengths		Bond angles	
		RMSZ	#$\|Z\|$ >5	RMSZ	#$\|Z\|$ >5
1	A	0.64	0/2242	0.76	1/3060 (0.0%)
1	B	0.68	0/2309	0.78	1/3147 (0.0%)
All	All	0.66	0/4551	0.77	2/6207 (0.0%)

There are no bond length outliers.

All (2) bond angle outliers are listed below:

Mol	Chain	Res	Type	Atoms	Z	Observed(^{o})	Ideal(^{o})
1	A	248	ASP	CB-CG-OD1	5.43	123.19	118.30
1	B	187	ASP	CB-CG-OD1	5.36	123.13	118.30

There are no chirality outliers.

There are no planarity outliers.

5.2 Too-close contacts (i)

In the following table, the Non-H and H(model) columns list the number of non-hydrogen atoms and hydrogen atoms in the chain respectively. The H(added) column lists the number of hydrogen atoms added and optimized by MolProbity. The Clashes column lists the number of clashes within the asymmetric unit, whereas Symm-Clashes lists symmetry-related clashes.

Mol	Chain	Non-H	H(model)	H(added)	Clashes	Symm-Clashes
1	A	2194	0	2020	21	0
1	B	2255	0	2141	18	0
2	A	35	0	0	2	0
2	B	35	0	0	0	0
3	A	78	0	0	3	0
3	B	140	0	0	5	0
All	All	4737	0	4161	37	0

CONFIDENTIAL VALIDATION REPORT

The all-atom clashscore is defined as the number of clashes found per 1000 atoms (including hydrogen atoms). The all-atom clashscore for this structure is 4.

All (37) close contacts within the same asymmetric unit are listed below, sorted by their clash magnitude.

Atom-1	Atom-2	Interatomic distance (Å)	Clash overlap (Å)
1:B:4:ARG:H	1:B:299:GLN:HE22	1.28	0.82
1:A:4:ARG:H	1:A:299:GLN:HE22	1.31	0.78
1:A:145[A]:CYS:SG	2:A:501:7XB:C07	2.75	0.75
1:A:186:VAL:H	1:A:192:GLN:HE22	1.37	0.73
1:A:28:ASN:ND2	1:A:120:GLY:H	1.89	0.69
1:A:87:LEU:CD2	1:A:89:LEU:HD21	2.22	0.69
1:B:288:GLU:HB2	3:B:611:HOH:O	1.93	0.67
1:B:221:ASN:HD22	1:B:270:GLU:HG3	1.60	0.67
1:A:110:GLN:HG3	3:A:635:HOH:O	1.96	0.66
1:B:64:HIS:HD2	3:B:604:HOH:O	1.79	0.65
1:B:145[A]:CYS:SG	1:B:164:HIS:O	2.58	0.62
1:A:138:GLY:H	1:A:172:HIS:HD2	1.47	0.60
1:A:4:ARG:H	1:A:299:GLN:NE2	1.99	0.60
1:A:95:ASN:HB3	1:A:98:THR:OG1	2.02	0.60
1:B:51:ASN:OD1	1:B:188:ARG:NH1	2.36	0.58
1:B:221:ASN:ND2	1:B:270:GLU:HG3	2.21	0.55
1:B:53:ASN:C	1:B:53:ASN:HD22	2.10	0.55
1:A:28:ASN:HD22	1:A:120:GLY:H	1.53	0.55
1:A:285:ALA:HB3	1:B:285:ALA:HB3	1.89	0.54
1:B:41:HIS:HE1	3:B:587:HOH:O	1.91	0.53
1:A:226:THR:HG22	3:A:649:HOH:O	2.12	0.49
1:B:7:ALA:HA	1:B:127:GLN:OE1	2.13	0.49
1:A:247:VAL:HG22	1:A:261:VAL:HG11	1.95	0.48
1:A:4:ARG:N	1:A:299:GLN:HE22	2.07	0.48
1:B:83:GLN:NE2	1:B:178:GLU:HB3	2.28	0.48
1:A:128:CYS:HB3	1:B:4:ARG:HH22	1.80	0.46
1:A:226:THR:CG2	3:A:649:HOH:O	2.62	0.46
1:B:138:GLY:H	1:B:172:HIS:HD2	1.62	0.46
1:B:45:THR:O	1:B:49:MET:HG3	2.16	0.45
1:A:87:LEU:CD2	1:A:89:LEU:CD2	2.94	0.45
1:A:87:LEU:HD21	1:A:89:LEU:HD21	1.97	0.44
1:A:165:MET:CE	1:A:187:ASP:HA	2.47	0.44
1:B:127:GLN:NE2	3:B:506:HOH:O	2.49	0.44
1:B:221:ASN:ND2	1:B:270:GLU:CG	2.81	0.43
1:A:207:TRP:CE2	1:A:288:GLU:HB3	2.56	0.41
1:A:165:MET:HE1	2:A:501:7XB:F30	2.11	0.41
1:B:64:HIS:CD2	3:B:604:HOH:O	2.63	0.40

CONFIDENTIAL VALIDATION REPORT

There are no symmetry-related clashes.

5.3 Torsion angles (i)

5.3.1 Protein backbone (i)

In the following table, the Percentiles column shows the percent Ramachandran outliers of the chain as a percentile score with respect to all X-ray entries followed by that with respect to entries of similar resolution.

The Analysed column shows the number of residues for which the backbone conformation was analysed, and the total number of residues.

Mol	Chain	Analysed	Favoured	Allowed	Outliers	Percentiles	
1	A	293/311 (94%)	284 (97%)	8 (3%)	1 (0%)	41	37
1	B	301/311 (97%)	291 (97%)	10 (3%)	0	100	100
All	All	594/622 (96%)	575 (97%)	18 (3%)	1 (0%)	47	44

All (1) Ramachandran outliers are listed below:

Mol	Chain	Res	Type
1	A	154	TYR

5.3.2 Protein sidechains (i)

In the following table, the Percentiles column shows the percent sidechain outliers of the chain as a percentile score with respect to all X-ray entries followed by that with respect to entries of similar resolution.

The Analysed column shows the number of residues for which the sidechain conformation was analysed, and the total number of residues.

Mol	Chain	Analysed	Rotameric	Outliers	Percentiles	
1	A	222/268 (83%)	216 (97%)	6 (3%)	44	46
1	B	239/268 (89%)	235 (98%)	4 (2%)	60	65
All	All	461/536 (86%)	451 (98%)	10 (2%)	52	55

All (10) residues with a non-rotameric sidechain are listed below:

Mol	Chain	Res	Type
1	A	1	SER
1	A	27	LEU

Continued on next page...

CONFIDENTIAL VALIDATION REPORT

Continued from previous page...

Mol	Chain	Res	Type
1	A	87	LEU
1	A	263	ASP
1	A	267	SER
1	A	286	LEU
1	B	27	LEU
1	B	53	ASN
1	B	81	SER
1	B	87	LEU

Sometimes sidechains can be flipped to improve hydrogen bonding and reduce clashes. All (16) such sidechains are listed below:

Mol	Chain	Res	Type
1	A	28	ASN
1	A	84	ASN
1	A	164	HIS
1	A	172	HIS
1	A	180	ASN
1	A	192	GLN
1	A	299	GLN
1	B	41	HIS
1	B	53	ASN
1	B	63	ASN
1	B	64	HIS
1	B	164	HIS
1	B	172	HIS
1	B	180	ASN
1	B	273	GLN
1	B	299	GLN

5.3.3 RNA (i)

There are no RNA molecules in this entry.

5.4 Non-standard residues in protein, DNA, RNA chains (i)

There are no non-standard protein/DNA/RNA residues in this entry.

5.5 Carbohydrates (i)

There are no monosaccharides in this entry.

CONFIDENTIAL VALIDATION REPORT

5.6 Ligand geometry ⓘ

2 ligands are modelled in this entry.

In the following table, the Counts columns list the number of bonds (or angles) for which Mogul statistics could be retrieved, the number of bonds (or angles) that are observed in the model and the number of bonds (or angles) that are defined in the Chemical Component Dictionary. The Link column lists molecule types, if any, to which the group is linked. The Z score for a bond length (or angle) is the number of standard deviations the observed value is removed from the expected value. A bond length (or angle) with $|Z| > 2$ is considered an outlier worth inspection. RMSZ is the root-mean-square of all Z scores of the bond lengths (or angles).

Mol	Type	Chain	Res	Link	Bond lengths			Bond angles		
					Counts	RMSZ	$\#\|Z\| > 2$	Counts	RMSZ	$\#\|Z\| > 2$
2	7XB	A	501	-	30,37,37	2.01	8 (26%)	42,53,53	2.09	11 (26%)
2	7XB	B	401	-	30,37,37	1.94	9 (30%)	42,53,53	1.88	7 (16%)

In the following table, the Chirals column lists the number of chiral outliers, the number of chiral centers analysed, the number of these observed in the model and the number defined in the Chemical Component Dictionary. Similar counts are reported in the Torsion and Rings columns. '-' means no outliers of that kind were identified.

Mol	Type	Chain	Res	Link	Chirals	Torsions	Rings
2	7XB	A	501	-	-	6/18/18/18	0/3/3/3
2	7XB	B	401	-	-	7/18/18/18	0/3/3/3

All (17) bond length outliers are listed below:

Mol	Chain	Res	Type	Atoms	Z	Observed(Å)	Ideal(Å)
2	A	501	7XB	C10-N09	7.50	1.44	1.33
2	B	401	7XB	C10-N09	7.26	1.43	1.33
2	B	401	7XB	C12-N11	3.07	1.48	1.39
2	A	501	7XB	C07-N09	3.01	1.44	1.38
2	A	501	7XB	C12-N11	2.91	1.48	1.39
2	A	501	7XB	O04-C03	-2.83	1.17	1.23
2	B	401	7XB	C03-N02	2.51	1.46	1.34
2	A	501	7XB	C03-N02	2.39	1.45	1.34
2	B	401	7XB	O04-C03	-2.34	1.18	1.23
2	B	401	7XB	C05-N06	-2.33	1.44	1.49
2	B	401	7XB	O17-C16	2.24	1.42	1.38
2	B	401	7XB	C07-N09	2.22	1.42	1.38
2	B	401	7XB	C10-N11	2.22	1.45	1.36
2	A	501	7XB	C10-N11	2.18	1.45	1.36
2	A	501	7XB	O17-C16	2.12	1.42	1.38

Continued on next page...

CONFIDENTIAL VALIDATION REPORT

Continued from previous page...

Mol	Chain	Res	Type	Atoms	Z	Observed(Å)	Ideal(Å)
2	A	501	7XB	C24-N23	-2.08	1.44	1.48
2	B	401	7XB	C21-C16	2.03	1.42	1.38

All (18) bond angle outliers are listed below:

Mol	Chain	Res	Type	Atoms	Z	Observed(^{o})	Ideal(^{o})
2	A	501	7XB	C07-N09-C10	7.78	119.68	112.95
2	B	401	7XB	C07-N09-C10	7.36	119.31	112.95
2	B	401	7XB	O04-C03-C05	-5.16	111.63	120.04
2	A	501	7XB	C05-N06-C34	3.73	122.29	117.92
2	A	501	7XB	C25-C24-N23	-3.59	107.04	112.63
2	B	401	7XB	C05-C03-N02	3.52	122.11	116.27
2	A	501	7XB	C12-N11-C10	-3.36	118.76	128.05
2	A	501	7XB	C24-C25-C26	-3.16	115.35	120.25
2	A	501	7XB	C31-C29-C27	3.03	121.69	118.69
2	A	501	7XB	C33-C31-C29	-2.83	117.61	121.68
2	A	501	7XB	O04-C03-C05	-2.66	115.70	120.04
2	A	501	7XB	C14-C13-C12	2.63	124.23	121.25
2	B	401	7XB	C25-C24-N23	-2.56	108.66	112.63
2	B	401	7XB	C31-C29-C27	2.47	121.13	118.69
2	A	501	7XB	C24-C25-C33	2.31	123.84	120.25
2	A	501	7XB	F32-C31-C29	2.24	121.44	118.30
2	B	401	7XB	C33-C31-C29	-2.10	118.65	121.68
2	B	401	7XB	F32-C31-C33	2.10	122.78	118.61

There are no chirality outliers.

All (13) torsion outliers are listed below:

Mol	Chain	Res	Type	Atoms
2	A	501	7XB	C03-C05-N06-C07
2	B	401	7XB	N23-C24-C25-C33
2	A	501	7XB	N23-C24-C25-C33
2	B	401	7XB	N23-C24-C25-C26
2	A	501	7XB	N23-C24-C25-C26
2	B	401	7XB	C03-C05-N06-C07
2	A	501	7XB	O04-C03-C05-N06
2	B	401	7XB	O04-C03-C05-N06
2	A	501	7XB	N02-C03-C05-N06
2	B	401	7XB	N02-C03-C05-N06
2	B	401	7XB	C15-C16-O17-C18
2	A	501	7XB	C22-C12-N11-C10
2	B	401	7XB	C22-C12-N11-C10

CONFIDENTIAL VALIDATION REPORT

There are no ring outliers.

1 monomer is involved in 2 short contacts:

Mol	Chain	Res	Type	Clashes	Symm-Clashes
2	A	501	7XB	2	0

The following is a two-dimensional graphical depiction of Mogul quality analysis of bond lengths, bond angles, torsion angles, and ring geometry for all instances of the Ligand of Interest. In addition, ligands with molecular weight > 250 and outliers as shown on the validation Tables will also be included. For torsion angles, if less then 5% of the Mogul distribution of torsion angles is within 10 degrees of the torsion angle in question, then that torsion angle is considered an outlier. Any bond that is central to one or more torsion angles identified as an outlier by Mogul will be highlighted in the graph. For rings, the root-mean-square deviation (RMSD) between the ring in question and similar rings identified by Mogul is calculated over all ring torsion angles. If the average RMSD is greater than 60 degrees and the minimal RMSD between the ring in question and any Mogul-identified rings is also greater than 60 degrees, then that ring is considered an outlier. The outliers are highlighted in purple. The color gray indicates Mogul did not find sufficient equivalents in the CSD to analyse the geometry.

Ligand 7XB A 501

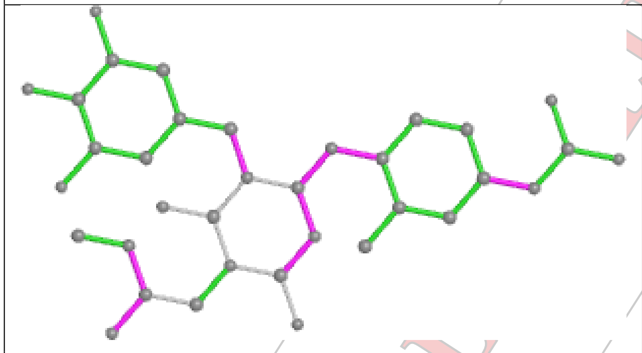

Bond lengths

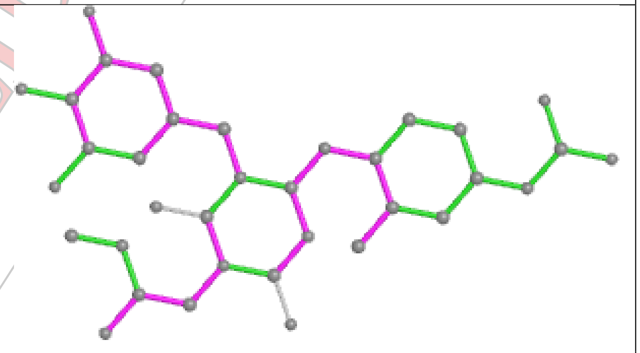

Bond angles

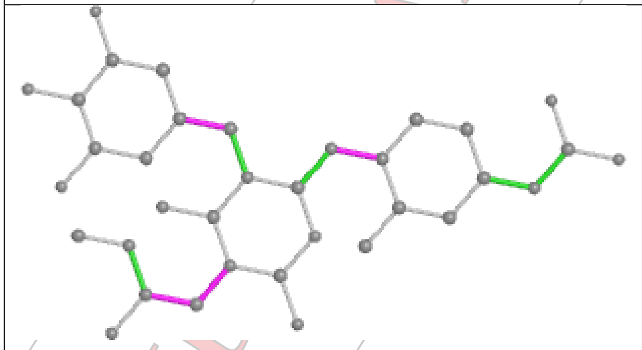

Torsions

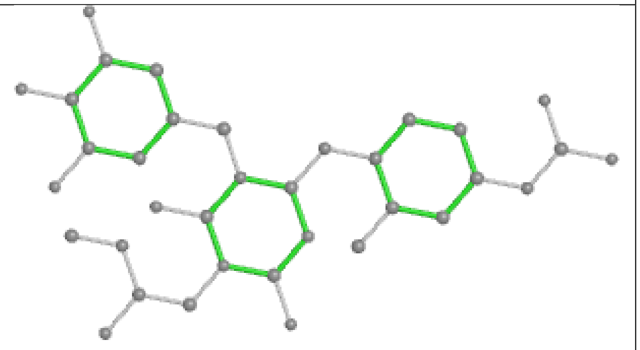

Rings

CONFIDENTIAL VALIDATION REPORT

Ligand 7XB B 401

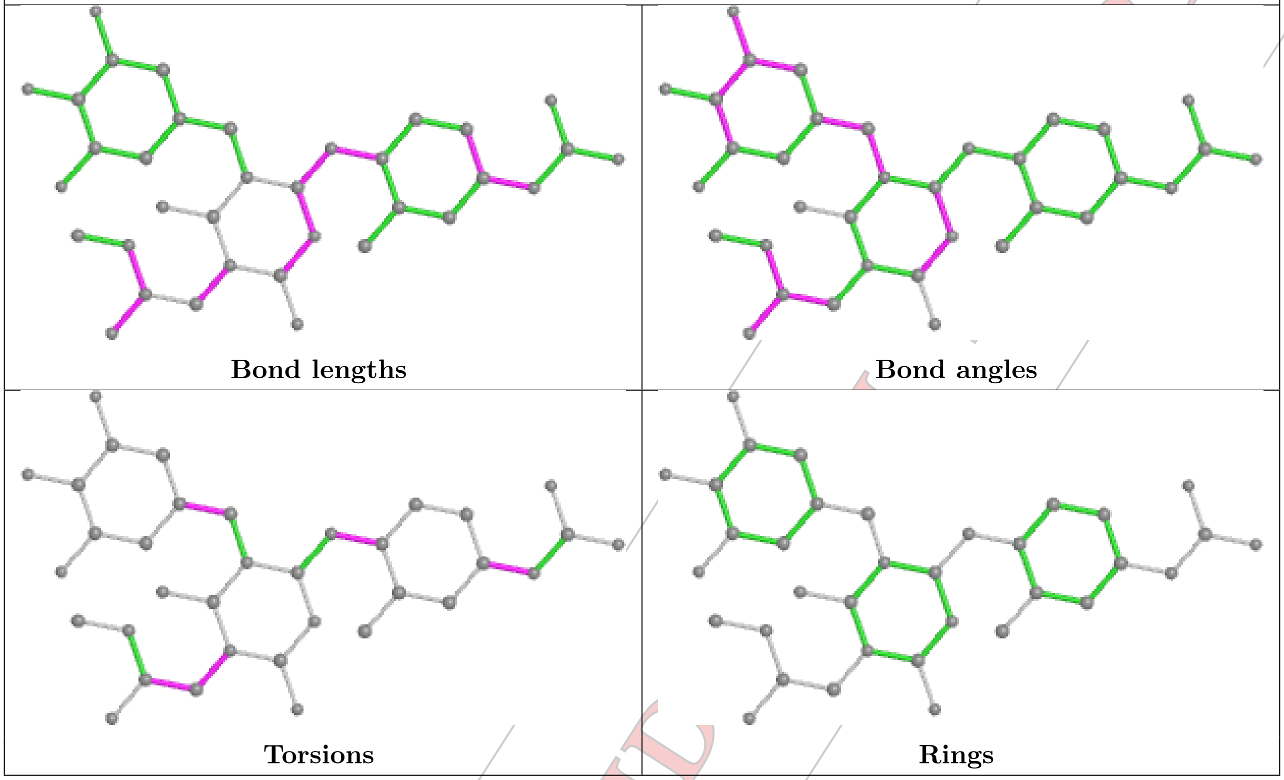

5.7 Other polymers (i)

There are no such residues in this entry.

5.8 Polymer linkage issues (i)

There are no chain breaks in this entry.

CONFIDENTIAL VALI

6 Fit of model and data (i)

6.1 Protein, DNA and RNA chains (i)

In the following table, the column labelled '#RSRZ> 2' contains the number (and percentage) of RSRZ outliers, followed by percent RSRZ outliers for the chain as percentile scores relative to all X-ray entries and entries of similar resolution. The OWAB column contains the minimum, median, 95th percentile and maximum values of the occupancy-weighted average B-factor per residue. The column labelled 'Q< 0.9' lists the number of (and percentage) of residues with an average occupancy less than 0.9.

Mol	Chain	Analysed	<RSRZ>	#RSRZ>2			OWAB(Å^2)	Q<0.9
1	A	298/311 (95%)	-0.09	3 (1%)	82	81	10, 23, 40, 53	2 (0%)
1	B	301/311 (96%)	-0.42	0	100	100	7, 16, 28, 46	0
All	All	599/622 (96%)	-0.25	3 (0%)	91	90	7, 18, 37, 53	2 (0%)

All (3) RSRZ outliers are listed below:

Mol	Chain	Res	Type	RSRZ
1	A	46	SER	2.8
1	A	73	VAL	2.8
1	A	154	TYR	2.6

6.2 Non-standard residues in protein, DNA, RNA chains (i)

There are no non-standard protein/DNA/RNA residues in this entry.

6.3 Carbohydrates (i)

There are no monosaccharides in this entry.

6.4 Ligands (i)

In the following table, the Atoms column lists the number of modelled atoms in the group and the number defined in the chemical component dictionary. The B-factors column lists the minimum, median, 95th percentile and maximum values of B factors of atoms in the group. The column labelled 'Q< 0.9' lists the number of atoms with occupancy less than 0.9.

Mol	Type	Chain	Res	Atoms	RSCC	RSR	B-factors(Å^2)	Q<0.9
2	7XB	A	501	35/35	0.89	0.16	20,29,42,51	0
2	7XB	B	401	35/35	0.89	0.13	15,22,34,38	0

CONFIDENTIAL VALIDATION REPORT

The following is a graphical depiction of the model fit to experimental electron density of all instances of the Ligand of Interest. In addition, ligands with molecular weight > 250 and outliers as shown on the geometry validation Tables will also be included. Each fit is shown from different orientation to approximate a three-dimensional view.

Electron density around 7XB A 501:

$2mF_o$-DF_c (at 0.7 rmsd) in gray
mF_o-DF_c (at 3 rmsd) in purple (negative)
and green (positive)

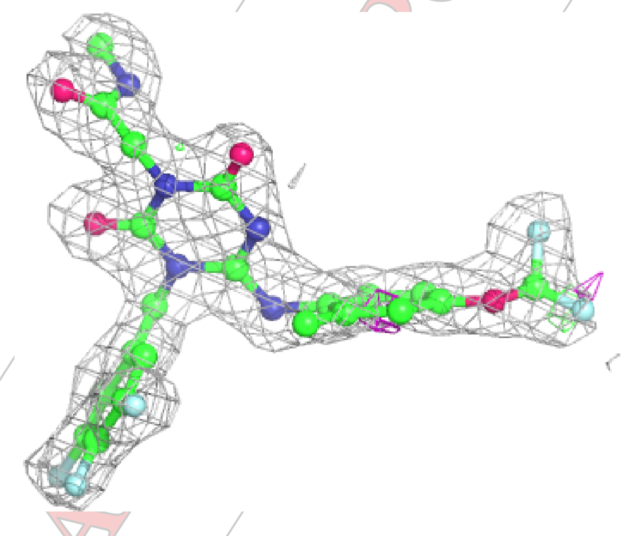

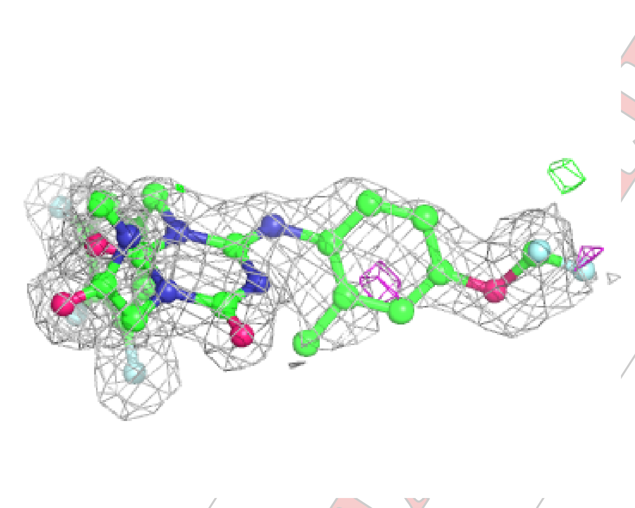

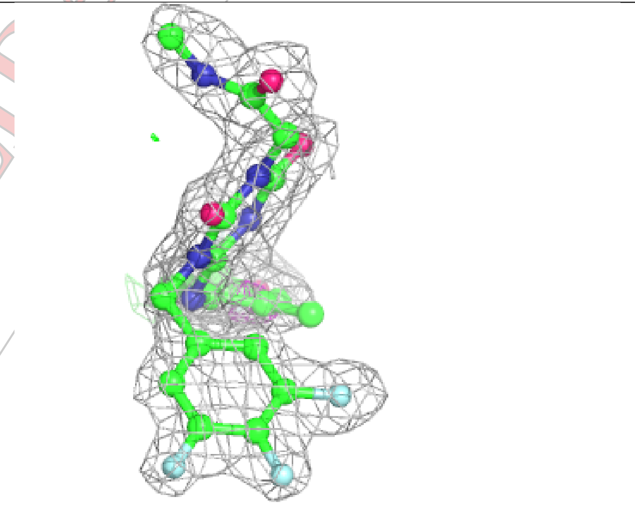

Electron density around 7XB B 401:

$2mF_o$-DF_c (at 0.7 rmsd) in gray
mF_o-DF_c (at 3 rmsd) in purple (negative)
and green (positive)

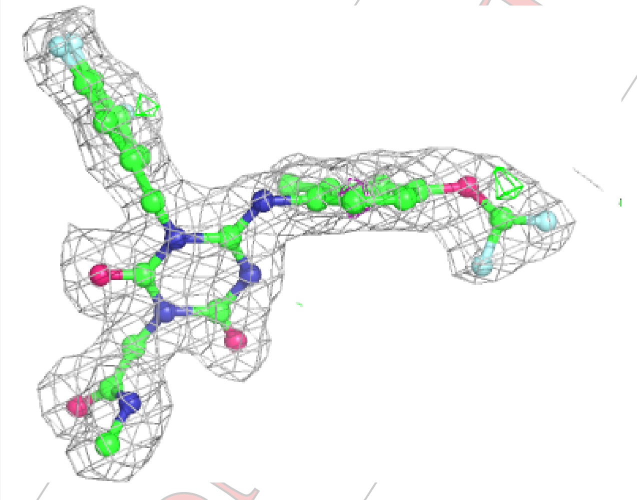

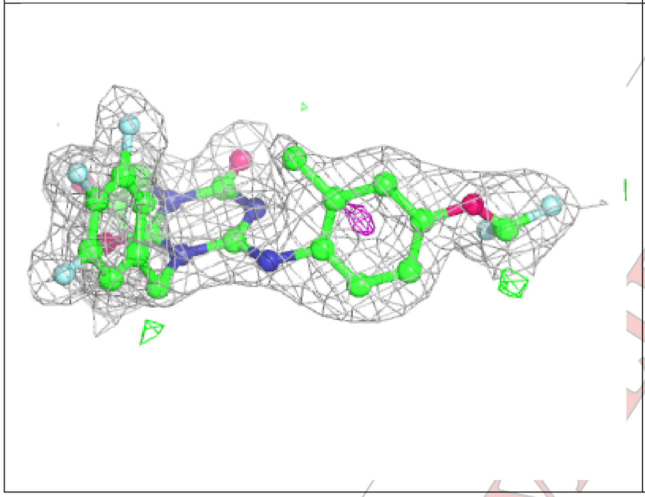

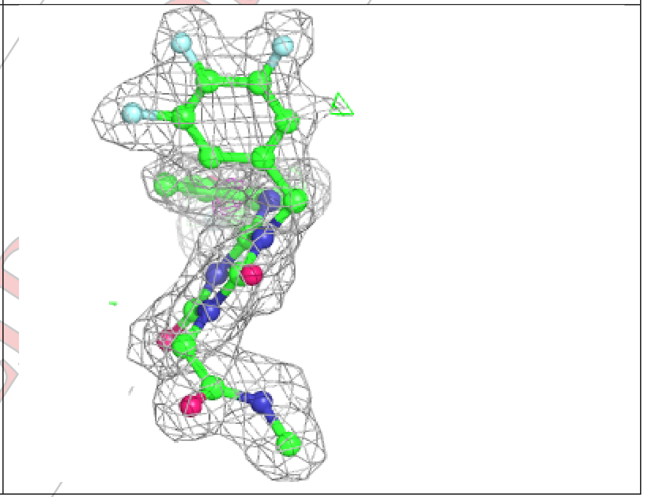

6.5 Other polymers (i)

There are no such residues in this entry.

CONFIDENTIAL
